# Biofilm and sediment phases as key components of microbial community dynamics within secondary drinking water distribution systems

**DOI:** 10.1186/s12866-026-05149-7

**Published:** 2026-05-16

**Authors:** Soledad Martínez, María Pía Cerdeiras, Isabel Douterelo, Umer Zeeshan Ijaz

**Affiliations:** 1 Microbiology Area, Water Analysis Unit, Faculty of Chemistry, University of the Republic, Montevideo, Uruguay; 2 Graduate Program in Chemistry, Faculty of Chemistry, University of the Republic, Montevideo, Uruguay; 3 Department of Biosciences, Faculty of Chemistry, University of the Republic, Montevideo, Uruguay; 4 School of Mechanical, Aerospace, and Civil Engineering, The University of Sheffield, Sheffield, UK; 5 Infrastructure and Environment Research Division, James Watt School of Engineering, University of Glasgow, Glasgow, UK

**Keywords:** Biofilms, Drinking water storage tanks, Microbial succession, Phase-resolved microbiome, Secondary drinking water distribution systems

## Abstract

**Background:**

Secondary drinking water distribution systems (SDWDS), particularly rooftop storage tanks, are critical components of water supply infrastructure in many regions, yet the ecological processes governing microbial community development within these systems remain poorly characterized. Here we present a year-long, phase-resolved metagenomic study of an operational full-scale SDWDS in Uruguay to assess how environmental conditions and surface materials are associated with microbiome dynamics across bulk water, biofilm and sediment phases. We integrated amplicon sequencing, whole-genome sequencing (WGS) metagenomics, culture-based microbiology and physicochemical analyses over a one-year period.

**Results:**

Microbial communities associated with biofilm and sediment phases consistently exhibited higher richness and diversity than bulk water, with marked seasonal variation. Biofilms formed on concrete and polyethylene surfaces followed distinct successional trajectories, indicating material-associated patterns in community development. Seasonal increases in temperature were associated with greater similarity in community composition across phases, while functional richness remained comparatively stable over time. Functional pathways related to energy production, stress response, and antibiotic resistance showed phase- and time-dependent enrichment, particularly in mature biofilms. Across the system, Proteobacteria, Actinobacteriota, and Bacteroidota were persistent taxa. Temperature and pH were the primary variables associated with temporal shifts in water-phase microbial communities, with chlorine residuals contributing to additional variation.

**Conclusions:**

Together, these findings provide in situ ecological insight into microbial succession and phase-specific community dynamics in drinking water storage systems, highlighting the importance of long-term observations in real-world engineered environments.

**Supplementary Information:**

The online version contains supplementary material available at 10.1186/s12866-026-05149-7.

## Background

Secondary drinking water distribution systems (SDWDS) are widely used in developing regions and refer to building-scale water networks composed of pipes, pumps, storage tanks that ensures an adequate water supply and pressure. Rooftop storage tanks are particularly common in these systems, guaranteeing water availability when supply pressure is insufficient [1–3]. In Latin America, SDWDS use is widespread and continues to expand, even in single-story houses or rural areas, to improve water pressure or for water storage purposes [4].

In Uruguay, most households are connected to the main distribution system, yet secondary storage tanks are widely employed as part of SDWDS. These systems create distinct engineered environments in which water is stored for extended periods, often under warm climatic conditions. Prolonged retention times—often caused by mismatches between tank size and household water demand—combined with high summer temperatures favour disinfectant loss, biofilm development on tank surfaces and the accumulation of sediments [1–3, 5–9]. These conditions support the establishment of complex microbial communities capable of harbouring opportunistic pathogens, turning storage tanks into reservoirs and amplifiers of microbial risk [1–3, 8, 10].

Despite their ubiquity, storage tanks remain largely overlooked in microbiological monitoring frameworks, which also typically focus on bulk water quality [11]. Although several studies have assessed bulk water quality and safety in relation to tank material, cleaning frequency, and retention times [12–15], it is known that up to 98% of microorganisms within drinking water distribution systems are located in the biofilms attached to inner wall surfaces or in the sediments of the tanks [7, 16, 17]. Biofilm-associated microorganisms display enhanced persistence and resistance [18], yet their ecology within SDWDS storage tanks is poorly characterized, particularly in long-term, operational settings. This knowledge gap is particularly evident in Latin America, where empirical data on microbial risks in building-scale drinking water infrastructure are scarce [4].

Here, we address these gaps by presenting a long-term, phase-resolved characterization of the microbiome within an operational SDWDS in Montevideo, Uruguay. To begin addressing these issues, we used various techniques to characterize the entire system (water, sediments, and biofilms) at multiple times between tank cleanings. We expected that, from a microbiological perspective, water quality would deteriorate over time since cleaning, and sediments and biofilms would play a key role in shaping the tank’s water quality.

We combined 16 S rRNA amplicon sequencing, whole-genome shotgun metagenomics, culture-based microbiology, and physicochemical analyses to investigate how biofilms formed on concrete and high-density polyethylene surfaces develop over time and influence microbial community dynamics across water, biofilm and sediment phases. By integrating taxonomic composition, functional profiles and environmental parameters, this study provides new insight into the ecological mechanisms—material-dependent interactions, environmental drivers and temporal processes—that shape biofilm succession in drinking water storage environments. These findings advance understanding of environmental microbiomes in engineered water systems and support improved monitoring and risk management strategies for SDWDS.

## Methods

### Experimental SDWS and coupons design

The study was conducted in an operational SDWDS [19] consisting of three interconnected reinforced concrete tanks with a total capacity of 31,500 L located outside of a three-story University building in the city centre of Montevideo, Uruguay.

**Fig. 1:**
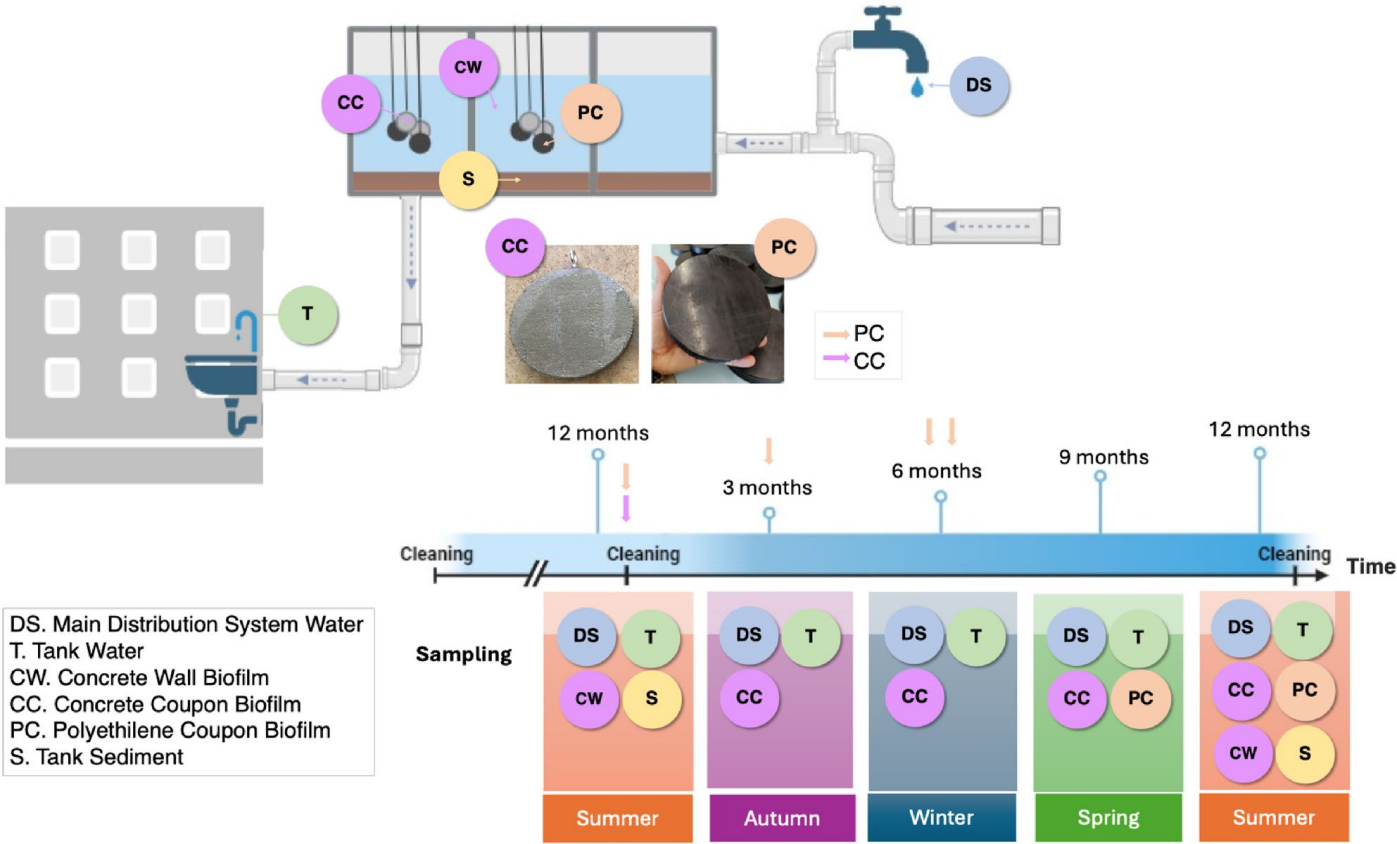
Sampling regime highlighting different compartments, strategies, and timepoints at which the data is collected

To assess biofilm formation, 15 cm circular coupons made of two different materials (concrete and polyethylene) were designed: (i) using the same materials and methods as the interior surfaces of the tank walls, floor, and ceiling (3:1 sand and Portland cement mortar, polished with pure Portland cement) and (ii) polyethylene complying with Uruguayan regulations for tank construction materials [19]. No anti-corrosion coating or additional treatment has been applied to the inner surfaces of the tank or coupons. A total of 22 disinfected reinforced concrete coupons and 17 polyethylene coupons were fully submerged in the water tank, suspended from the lids of two of the three interconnected tanks. Figure 1 shows a sketch of the experimental setup of the coupons in the tanks. The set of 22 concrete and 3 polyethylene coupons were placed immediately after the tank annual cleaning (March 2022) and prior to the disinfection of the entire unit with a 50 a 100 mg/l sodium hypochlorite solution [20]. The remaining polyethylene coupons were placed in the tank in May and September 2022 due to delays in coupons production.

### Sampling water, biofilms and sediments

Sampling of the coupons, water from the tank and main distribution system was conducted every 3 months over a year, form March 2022 to March 2023. Biofilm samples from the concrete walls and sediment were collected immediately prior to the annual cleaning (i.e. 12 months old biofilms and sediments) when the tank was emptied. These samples were referred to as “Pre” (developed over a year between March 2021–March 2022, sampled in the summer 2022) and “12 months” (from March 2022–March 2023, sampled in the summer 2023).

On each sampling date (Fig. 1), 4 coupons of each material were removed and transported to the lab in 2 L sterile sample bags (Whirl-Pak^®^, USA). Additionally, before each tank cleaning, sediment and biofilm samples from the walls were collected. Biofilms were removed from surfaces by brushing a 10 cm² surface with a sterile brush and resuspended in 25 ml of sterile Phosphate Buffer Saline (PBS).

Sediment samples were collected in a 2 L sterile sample bags (Whirl-Pak^®^, USA) from a sediment column from the bottom of the tank when the tank was nearly empty, immediately before the cleaning procedure.

Water samples were collected from taps upstream (water from the main distribution system) and downstream the tank (tank water). A subsample of 300 ml of water from these taps were collected to perform culture-based analysis and 6 replicates of 2 L were collected for DNA extraction on sterile bags (Whirl-Pak^®^, USA) containing sodium thiosulfate to quench the chlorine present [21].

Several environmental and physicochemical parameters were measured in the water at the time of sampling: residual free chlorine (HACH^®^ Pocket II colorimeter), turbidity (HACH^®^ 2100Q turbidimeter), pH (OAKTON^®^ pHTestr pH meter) and temperature were measured for all water samples, along with ambient temperature [22–24]. These parameters were selected as routine monitoring indicators and early-warning parameters of water quality, in accordance with national drinking water regulations (UNIT 833:2008). Additionally, a water flow meter (M191383, GENEBRE, Spain) was installed upstream of the tank to monitor water consumption.

### Water microbial quality and safety parameters tests

All samples were analysed for all the mandatory parameters required by Uruguayan drinking water standards [11]. For sediment and biofilm samples, in the absence of an applicable standard, the four techniques were adapted accordingly. For water or sediment samples, detection of total coliforms and *E. coli* was performed on 100 ml of sample (for sediment, corresponding to 20 mg of dry weight) using the commercial chromogenic medium Colitag™ following manufacturer’s instructions [25]. Detection of *P. aeruginosa* was done on 10 ml (for sediment, corresponding to 0.20 mg of dry weight) using asparagine broth (BD DifcoTM, USA), acetamide broth (Merck, Germany) and cetrimide agar (BD Difco™, USA) [26, 27]. Heterotrophic plate count (HPC) was performed on R2A media (BD DifcoTM, USA) by duplicate pour plating with 1:10 serial dilutions [28]. For biofilm samples, an entire coupon (100 cm²) was brushed into 25 ml of sterile PBS buffer, and 1 ml was used for the analyses referred above.

### DNA extraction, 16 S rRNA amplicon and shotgun metagenomic sequencing

For DNA extraction, all samples (i.e. 2 L of water, 0.5 L of sediment suspension, and 100 cm² of biofilm suspension in PBS) were subjected to vacuum filtration using a sterile nitrocellulose membrane with a pore size of 0.22 μm (Sartorius™, USA) [29]. DNA extraction was performed using the DNeasy PowerLyzer PowerSoil^®^ kit (Qiagen, Germany) and DNA concentrations were determined using the Qubit dsDNA High Sensitivity kit on a QubitTM fluorometer (Invitrogen by Thermo ScientificTM, USA).

A total of 57 samples were sequenced using a 16 S rRNA amplicon approach, distributed as follows: 6 from water of the main distribution system; 17 from tank water; 16 from tank concrete-tank biofilm (coupons and tank wall); 9 from polyethylene-tank-coupons biofilm; 6 from tank sediment and of a mock community. Sequencing of the V3-V4 region (primers: 341 F (CCTACGGGNGGCWGAG) and 805R (GACTACHVGGGTATCTAATCC)) was conducted using Illumina MiSeq technology, following a 300 bp paired-end protocol with a desired depth of 100,000 reads per library, provided by Macrogen (www.dna.macrogen.com, Seoul, South Korea).

For shotgun metagenomic sequencing, libraries from 20 pooled samples, pooled to meet the minimum DNA requirement of 100ng, were prepared using the IDT xGenTM DNA Lib Prep EZ kit at the Oklahoma Medical Research Foundation Genomics Core (Oklahoma City, USA) according to the manufacturer’s protocol and sequenced on an Illumina NovaSeq S4 platform using a 150 bp paired-end protocol.

### Bioinformatics and statistical methods

#### 16 S rRNA amplicon sequencing

For amplicon samples (*n* = 57), a total of 4,182,655 reads were obtained. Abundance tables were obtained by constructing Operational Taxonomic Units (OTUs), a proxy for species level assignment, using a modified workflow [30] where, a 99% threshold was used. Amplicon sequence variants (ASVs) were initially inferred using the DADA2 algorithm [31]; however, the mean number of reads assigned to ASVs per sample (12,428) was lower than that obtained with the OTU-based VSEARCH pipeline, and therefore, the OTU dataset was retained for downstream analyses. Briefly, preprocessing included quality trimming with Sickle [32], error correction with BayesHammer [33], and read merging with PANDASeq [34], resulting in 4,058,250 reads (*n* = 57). OTU construction utilized the VSEARCH pipeline [35] with *de novo* and reference-based chimera filtering against the SILVA gold database (https://www.mothur.org/w/images/f/f1/Silva.gold.bacteria.zip). Taxonomy was assigned using the SILVA SSU Ref NR v.138 [36] database within QIIME2 [37], which also generated a rooted phylogenetic tree. PICRUSt2 [38] was employed to predict KEGG Orthologs and MetaCyc pathways. The final dataset included a 57 × 5,966 OTU abundance table with the summary statistics of OTUs per sample as [1st Quartile: 43,183; Median: 49,010; Mean: 54,206; 3rd Quartile: 69,061; and Max: 84,220], supplemented with KEGG Ortholog (*n* = 57 x *P* = 10,543) and MetaCyc pathway (*n* = 57 x *P* = 489) abundance tables.

#### Shotgun metagenomics

For 21 metagenomic samples, the adapter-trimmed reads underwent quality filtering using Sickle [32] and assembly with Megahit, producing 391,236 contigs, a total of 1,453,073,008 base pairs (bp), maximum of 1,627,611 bp, average length of 3,714 bp, and an N50 score of 6,762 bp. Then contigs were binned using MetaWRAP pipeline [39] with three different binning algorithms i.e. metabat2 (381 bins) [40], maxbin2 (323 bins) [41], and CONCOCT (324 bins) [42]. Within MetaWRAP framework, the bins from the three binners were consolidated together to give a final set of 183 bins [Metagenome Assembled Genomes (MAGs)], with a mean genome completion of 77.87% and a mean contamination of 4.020% (CheckM [43], . Taxonomy was assigned using GTDB-TK [44] database, and for functional annotation we employed METABOLIC pipeline [45], integrating KEGG [46], TIGRfam [47], Pfam [48], custom hidden Markov model (HMM) databases [49], dbCAN2 [50], and MEROPS [51]. MAG phylogeny was reconstructed using GToTree [52], with coverage tables generated via CoverM (https://github.com/wwood/CoverM*).*

Further details on the bioinformatics methods used in this study are available in Supplementary_Information.docx.

#### Statistical analysis

Statistical analyses were performed in R (v 4.4.2) using the data generated from bioinformatics, as well as metadata associated with the study. Typically, R packages as Vegan [53] and phyloseq [54] were used for analyses. For the 16 S rRNA dataset, samples with > 5000 reads were selected, and removed typical contaminants such as *Mitochondria* and *Chloroplasts*, as well as any Operational Taxonomic Units (OTUs) that were unassigned at all levels, as per recommendations given at https://docs.qiime2.org/2022.8/tutorials/filtering/. For shotgun metagenomics, > 50% complete and < 10% contaminated MAGs were used (dismissing one mock community sample which was used as a quality control), resulting in a final table of 20 samples with 148 MAGs abundances.

Detailed information on statistical methods and models can be found in Supplementary_Information.docx.

## Results and discussion

### Spatial and temporal diversity trends

**Fig. 2:**
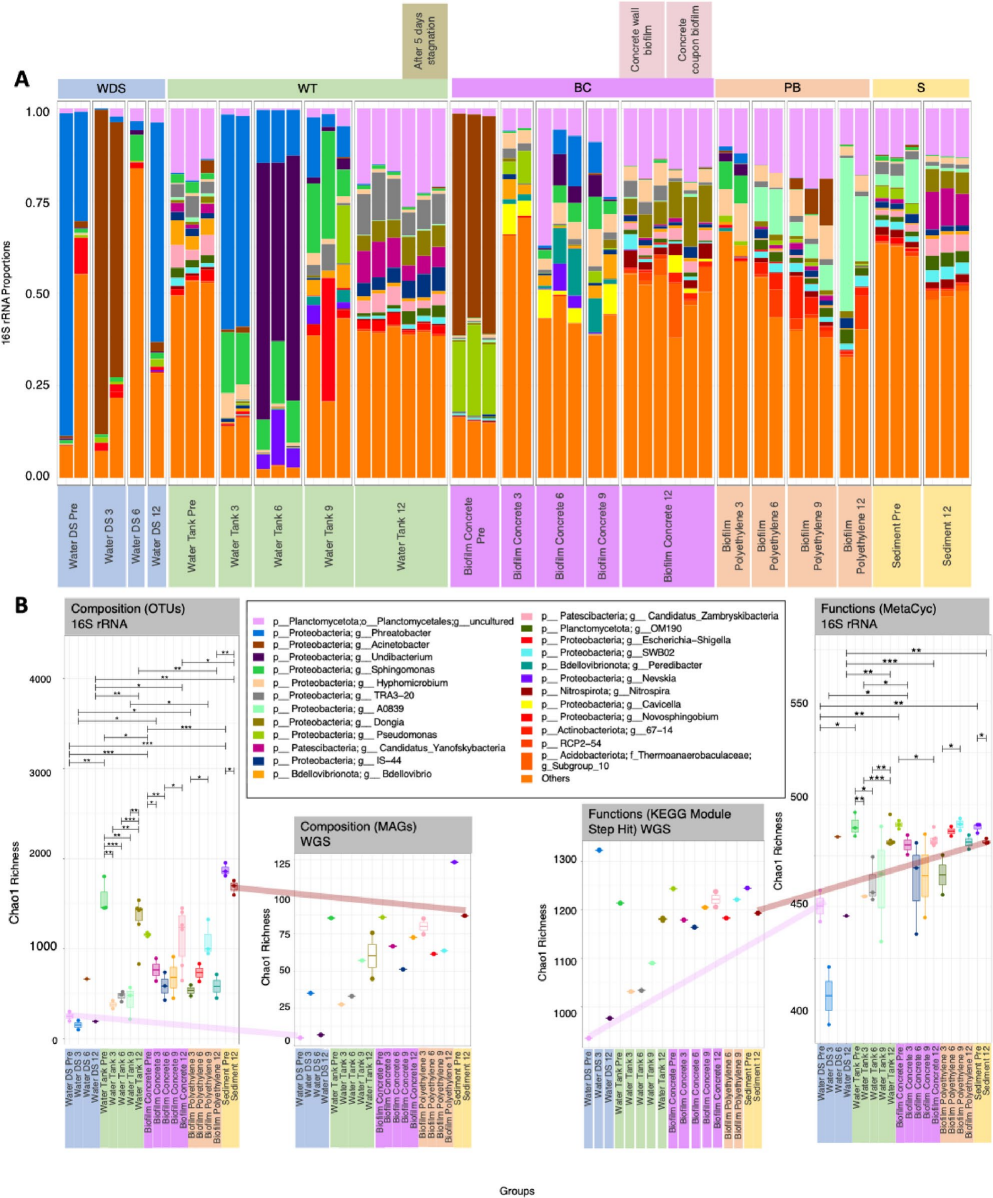
(**A**) Top 25 most abundant genera across the 16 S rRNA dataset (OTUs collated at genus level based on SILVA SSU Ref NR database release v.138 taxonomy) with the key provided below the barplots. (**B**) Chao 1 Richness comparison of 16 S rRNA based OTUs, and MAGs returned from WGS on the left. The comparison of functions (MetaCyc pathway abundances returned by PICRUSt2 software on OTUs, and KEGG Sub module abundances returned by METABOLIC software for MAGs) is shown on the right. The lines in each panel connect categories where the values are significantly different according to ANOVA with significance values as: * *p* < 0.05, ** *p* < 0.01, or *** *p* < 0.001. The thick lines across panels provide a visual cue to compare how both 16 S rRNA based amplification and WGS resulted in similar alpha diversity trends

A year-long sampling campaign, conducted immediately after tank cleaning and continuing until the next cleaning, captured microbial community dynamics across 3 SDWDS phases: bulk water (inlet water from the main system (WDS) and tank water (WT)), biofilm (formed over concrete (BC) and polyethylene (BP)), and sediment (S). Both 16 S-rRNA OTU and genome-resolved (MAG) approaches revealed similar α-diversity patterns (Fig. 2B and Supplementary Fig. S1), confirming the robustness of our findings.

Sediment and biofilm phases consistently exhibited higher richness than bulk water (Fig. 2B). Peak richness occurred in tank water, concrete biofilm, and sediment twelve months post-cleaning, with a water richness significant increment from 3 to 9 to 12 months (ANOVA *p* < 0.05, Fig. 2B). A similar pattern was observed in concrete biofilms, where 12-month-old biofilms were significantly richer than 6-month-old ones (ANOVA *p* < 0.05, Fig. 2B). Moreover, summer tank water samples showed higher richness and diversity than those from the mains (ANOVA *p* < 0.01, Fig. 2B). This suggests that seasonal shifts in storage tanks are driven by prolonged residence times, reduced flow, and chlorine decay, processes that intensify with rising temperatures, similarly as previous reports in main DWDS [1, 2, 6, 7, 55–57].

Among biofilms, concrete biofilm richness increased with biofilm age (Fig. 2B), reflecting progressive colonization and the formation of a more complex community. In contrast, in polyethylene samples, richness increased from 3 to 9 months, followed by a decline at 12 months (ANOVA, *p* < 0.05, Fig. 2B and Supplementary Fig. S1). Significant differences in richness and diversity between concrete and polyethylene were observed only in 12- month-old biofilms, with BC showing higher Chao 1 and Shannon values (ANOVA, *p* < 0.01, Fig. 2B and Supplementary Fig. S1). These findings show how biofilm diversity and stabilization stage can vary by tank material type as it occurs on pipes [58, 59]. The porous and alkaline nature of the concrete can provide heterogeneous microhabitats and continuous mineral release (e.g., calcium), favouring a sustained diversification and delayed stabilization of microbial communities [9]. Conversely, polyethylene is smooth and hydrophobic, thus supporting rapid and stochastic initial microbial colonization but limiting long-term ecological succession, showing a “rise and fall” pattern [57], which explains the peak at 9 months followed by a decline.

### Community composition dynamics

**Fig. 3:**
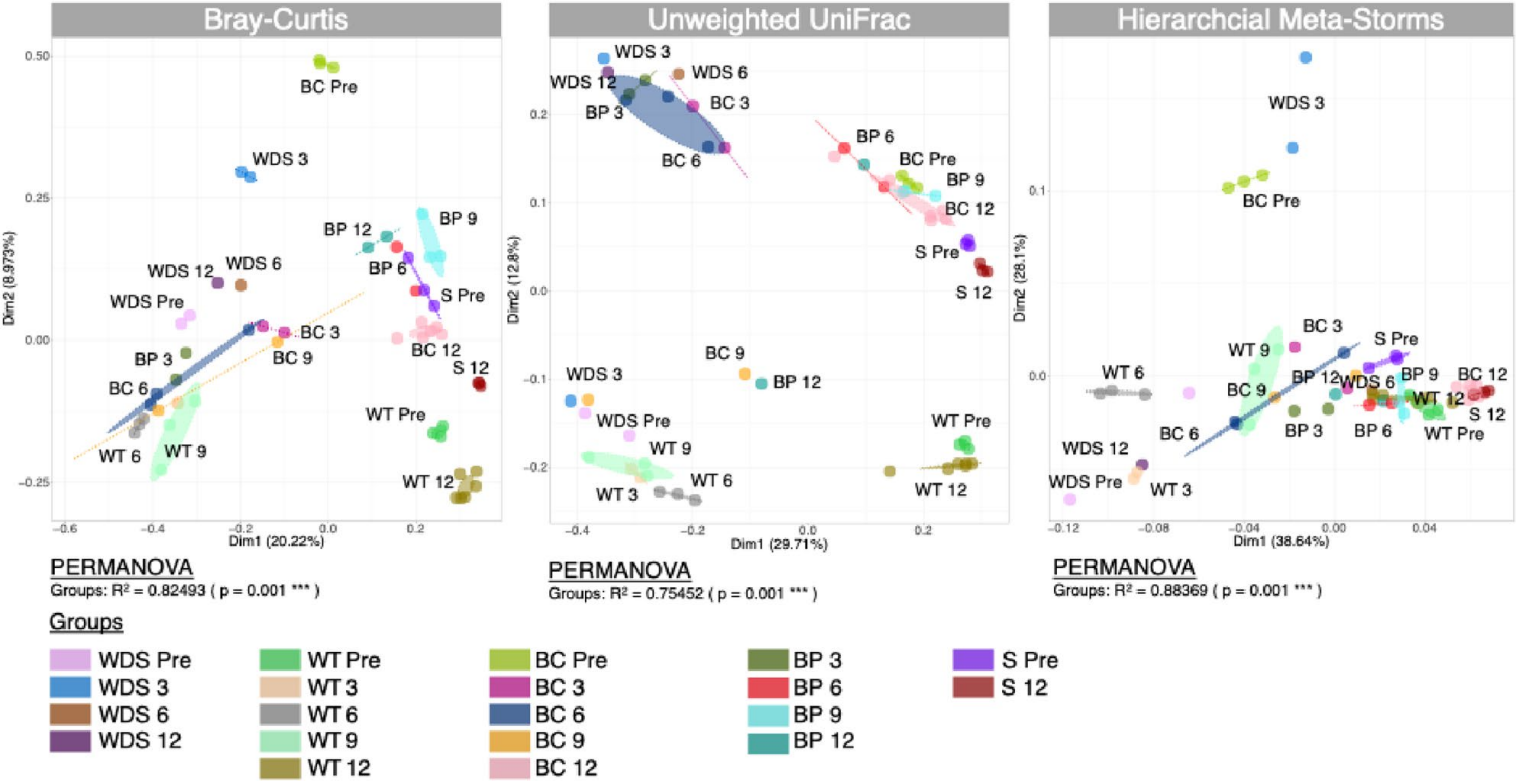
Phylogenetic tree of MAGs recovered via GToTree using 25 bacterial and archaeal specific SCG dataset. The tree also features G-C content, Quality index (genome completion – 5 x genome contamination), and Novelty (represented by phylogenetic gain (PG) values calculated using the GTDB toolkit. The outer rings show the heatmap of TSS + CLR normalised abundances collated for samples per each category. MAGs that were found to be significantly positively or negatively associated with time, based on CODA-LASSO regressions, are annotated with the last level of taxonomy as per GTDB toolkit. The detailed results are provided in Supplementary Figures S18-S20. The legends are as follows: WT+: MAGs positively associated for Water Tank; WT-: MAGs negatively associated for Water Tank; BC+: MAGs positively associated for Biofilm Concrete; and BC**-**: MAGs negatively associated for Biofilm Concrete

Microbial community composition varied markedly over time and across SDWDS phases, with strong seasonal patterns and distinct phase-specific taxa. Integration of 16 S rRNA-based taxonomic profiles with MAG-level resolution revealed overlapping yet distinct taxonomic patterns, highlighting the complementary information of both techniques (Fig. 2A and Fig. 3).

16 S rRNA analysis revealed clear seasonal shifts in dominant taxa in all the phases studied (WDS, WT, BC, BP and S), with the most abundant taxa generally consistent with those reported in other SDWDS [3, 56]. *Phreatobacter* dominated in WDS during summer (29.6–87.3%) and *Acinetobacter* became predominant in autumn (69.0-87.9%), whereas *Sphingomonas* (6.9%), Planctomycetales_uncultured order (3.5%), and *Phreatobacter* (2.4%) become more prominent in winter. Tank water communities transition from Planctomycetales_uncultured (13.7–26.6%) and Burkholderiales TRA3.20 (3.1–12.9%) dominating in summer, to *Phreatobacte*r (57.0-58.2%) and *Sphingomonas* (13.9–16.4%) in autumn and *Undibacterium* (48.3–69.5%) in winter. Genome-resolved data corroborated these temporal patterns, showing a decline of Rhizobiales_UBA4765 (often misclassified as *Phreatobacter* in amplicon data [60]), and enrichment of Burkholderiales and Zambryskibacteraceae MAGs, especially in summer. These results confirm the seasonal community turnover and reinforce the taxonomic resolution benefits of integrating genome-resolved data​.

Concrete biofilms transitioned from early communities dominated by Planctomycetales_uncultured order (5.4–6.0%) and the genera *Hyphomicrobium* (3.5–4.2%) and *Pseudomonas* (3.3–8.8%), to more mature assemblages by 12 months, enriched in Planctomycetales_uncultured (13.7–19.9%), the genera *Dongia* (4.3–13.4%) and *Hyphomicrobium* (2.0-6.1%) (Fig. 2A). After 12 months, concrete wall and concrete coupons biofilms both exhibited similar communities, reinforcing our initial idea that coupon surfaces effectively replicated wall conditions.

**Fig. 4:**
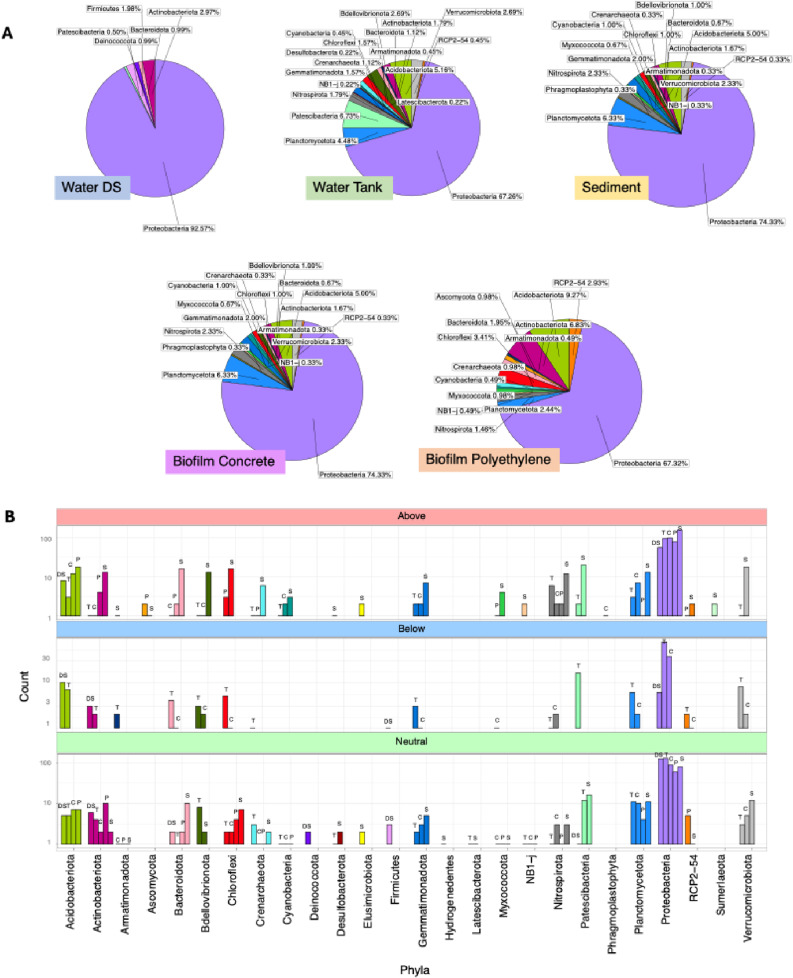
Beta diversity of 16 S rRNA OTUs represented by principal coordinate analysis (PCoA) plots with each axis showing the percentage variability explained by that axis, and where ellipses represent 95% confidence interval of the standard error for a given group. We have used three different distance measures: Bray-Curtis distance to show differences in composition, Unweighted UniFrac distance to show differences in phylogeny, and Hierarchical Meta-storms to show differences in metabolic function. PERMANOVA statistics utilising these distance measures are shown underneath to suggest if there are significant differences between the groups with R^2^

In contrast, concrete biofilms, formed over 12 months during the previous year (BC-Pre), were dominated by *Acinetobacter* (55.1–60.0%), *Pseudomonas* (18.9–24.7%) and Planctomycetales_uncultured (1.4–2.1%), showing a distinctive community (Fig. 4a, PERMANOVA: R² = 0.82493, *p* = 0.001). These results show that, beyond seasonal variations, biofilm communities do not return to the same composition after 12 months of formation in between cleanings. Fish and Boxall (2018) found that when biofilms were grown for 28 days under controlled conditions with different chlorine concentrations, the biofilm communities that developed under the same chlorine level were quite like each other [61]. This suggests that in the short term, one of the principal factors that shapes the composition of biofilms is chlorine concentration. In our long-term observations different water chemistry or environmental conditions, such as temperature or pH, influenced biofilm composition. In real distribution systems that are not maintained under controlled conditions, it is expected that biofilms will show greater heterogeneity over time and changing conditions.

Regarding material influence on biofilm growth, polyethylene consistently favoured members of the order Planctomycetales_uncultured (10.3–21.7%) across all sampling times. In more mature biofilms (12 months old) a higher abundance of *Rhizobiales_*A0839 (17.6–41.5%) was observed, while the genus *Hyphomicrobium* (2.5–10.1%) remained consistently present in all polyethylene samples (Fig. 2A). Thus, biofilms sampled in summer (6, 9, and 12 months of age) consistently share similar dominant taxa, suggesting that seasonality shapes community composition. This reflects both seasonal and material-specific selection processes, as material type is known to influence biofilm composition in main DWDS [58, 62, 63].

By the end of the cycle, during summer, tank water microbiomes diverged further from main distribution system water and resembled those of sediment and biofilm, indicating phase convergence within the tank (PERMANOVA R² ≈ 0.825, *p* = 0.001, Fig. 4)​. This overlap suggests environmental selection and microbial connectivity across phases, driven by shared adaptive traits within the tank environment [1, 2, 16, 56, 64].

### Functional shifts over time

Functional comparison (PICRUSt2 for OTUs and KEGG submodule for MAGs) (Fig. 2B) revealed that BC, BP and S exhibited the highest functional richness values. No clear temporal pattern in functional richness was observed for any of these phases. In contrast, tank water exhibited higher functional diversity during summer, potentially driven by the seasonal factors previously mentioned. Meanwhile, the lowest functional richness was observed in water from the mains, even in summer, when a significant difference between mains and tank water was detected (ANOVA, *p* < 0.01; Fig. 2B). PCoA based on Hierarchical Meta-Storms (Fig. 4C) revealed that most samples formed a distinct, large cluster, suggesting overall functional similarity across phases (PERMANOVA: R² = 0.88369, *p* = 0.001). These results indicate that, despite seasonal and material-driven shifts in taxonomic and phylogenetic composition, functional redundancy may occur, thus higher taxonomic diversity did not accompany higher potential functional diversity [64]. In microbial ecosystems, functionally similar but taxonomically distinct species can perform comparable roles in biogeochemical cycles [65].

Temporal trends in functional profiles were identified in tank water and concrete biofilm samples. CODA-LASSO regression models fitted to both datasets (PICRUSt2 predictions and METABOLIC’s KEGG submodules) achieved perfect predictive performance (R² = 1, *p* < 0.05), underscoring the robustness of the detected trends​ regardless of the analytical approach (Supplementary Fig. S18–S19). While PICRUSt2 trends were consistent with metagenomics, potential biases in environmental samples should be considered, and quantitative interpretations rely primarily on metagenomic data [66]. Nevertheless, the consistency between both approaches supports the value of presenting them together.

PICRUSt2 identified pathways linked to general metabolism, while WGS captured more specialized and stress-related functions. For example, tank water samples, PICRUSt2 predictions (Supplementary Fig. S18 A) identified ubiquinol-8 biosynthesis, homolactic fermentation and tetrahydrofolate biosynthesis as the most positively associated pathways with time. Using WGS data with KEGG modules (Supplementary Fig. S18B), we observed cytochrome bc1 complex respiratory unit, fatty acid biosynthesis and heme biosynthesis as the most positively associated pathways with time. These functions are linked to efficient energy generation, membrane synthesis, and redox processes, which indicates a more metabolically active, biofilm-influenced microbial community [67, 68]. This trend is consistent with increased residence time, reduced disinfectant pressure, higher temperatures, that promotes microbial regrowth and biofilm development.

In contrast to the more generalist functional profiles of water communities, concrete biofilms showed metabolic adaptations for survival, stress management, and community interactions. PICRUSt2-based predictions (Supplementary Fig. S19 A) revealed that pathways such as pyruvate fermentation to propanoate I, pyrimidine deoxyribonucleoside salvage, and polyamine biosynthesis II were the most positively associated with time. These pathways suggest that biofilm communities increasingly invest in metabolic adaptation (energy production, stress response) and cellular maintenance functions (maintaining genome integrity, regulating transcription, translation, cell growth) as biofilm formation progresses [67–70].

Similarly, WGS-based KEGG analysis (Supplementary Fig. S19 B) identified pathways associated with survival (xenobiotic degradation and transport) and resistance mechanisms [68], including the gamma-hexachlorocyclohexane transport system, Mce transport system and beta-lactam resistance, positively correlated with time in concrete biofilms highlighting the ubiquity of antibiotic resistance genes (ARGs) in biofilms and suggesting co-selection for resistance traits related to pollutant degradation and stress response, which may enhance biofilm persistence and resilience [71]. MAGs recovered from concrete biofilm samples that contain the M00627 module (beta-lactam resistance) are listed in Supplementary Table 1. Among them Gemmatimonadaceae showed a positive association with time and has previously been reported as part of pipe biofilms [72]. Notably, Mce proteins, which are known to participate in the virulence of pathogenic *Mycobacteria*, were also detected in nontuberculous Mycobacteria (NTM), involved in cell wall remodelling and lipid homeostasis [73]. NTM possessing AMR genes, conferring intrinsic resistance to key antibiotics, were detected as prevalent in disinfected DWDS [74]. The establishment of microorganisms which harbour improved survival mechanisms in drinking water systems raises public health concerns about biofilm persistence, potential antibiotic resistance, and the emergence of opportunistic pathogens. While it is known that DWDS can harbour emerging contaminants, including ARGs [74–77], this study is the first to report concrete water tank biofilms as real reservoirs of ARGs and associated them with time and biofilm maturity.

An additional finding was the negative association over time of the PilS-PilR two-component regulatory system pathway in biofilms, suggesting a decline in regulatory functions related to type IV pili, which are crucial for bacterial adhesion, surface motility, and biofilm development [78, 79]. The observed temporal decline, indicate a transition from an actively forming biofilm to a more mature and stable structure, where motility and surface attachment functions might become less necessary.

### Core microbiome composition across system phases

Core microbiome analysis was used to identify taxa consistently present across phases and time, offering insights into microbial stability, ecological function, and potential indicators for monitoring in SDWDS [80]. Core taxa were defined using occupancy models incorporating detection frequency and temporal consistency, with cutoffs optimized to balance diversity capture and redundancy (Supplementary Fig. S2).

A core microbiome set was iteratively constructed, stopping when the Bray–Curtis contribution increased by less than 2%, ensuring maximal diversity capture without redundant OTUs. Without taking any time-specific occupancy, the minimum occupancy (detection across samples) for core microbiome OTUs in the water from the main distribution system was 36%, for tank water was 51%, for sediment was almost 99%, for concrete biofilm 56% and for polyethylene biofilm 77%. These findings suggest that biofilms and sediments support more persistent microbial members than planktonic water phases [80], consistent with their greater structural stability and protective environmental conditions which led them as microbial reservoirs [7, 16]. In contrast, lower occupancy in water reflects a more dynamic microbial community, likely due to fluctuations in environmental conditions (e.g., flow, nutrient levels).

When considering time-specific occupancy, the taxonomy trees of the core microbiome revealed temporal and spatial variability across sample types. WT and both biofilm types (concrete and polyethylene) showed increasing phylogenetic complexity over time, with the most diverse core observed at 12 months post-cleaning (summer) (Supplementary Figures S4-S6). This likely reflects biofilm maturation and seasonal influences. In contrast, the main distribution system water exhibited dynamic tree structures throughout the monitoring period, likely due to fluctuating flow and shorter water residence times (Supplementary Fig. S3). In chlorinated main DWDS, planktonic microbial communities seem to be governed by more stochastic processes compared to biofilm [81]. However, our findings suggest that water from the main distribution system becomes comparatively more stochastic when contrasted storage tanks.

**Fig. 5:**
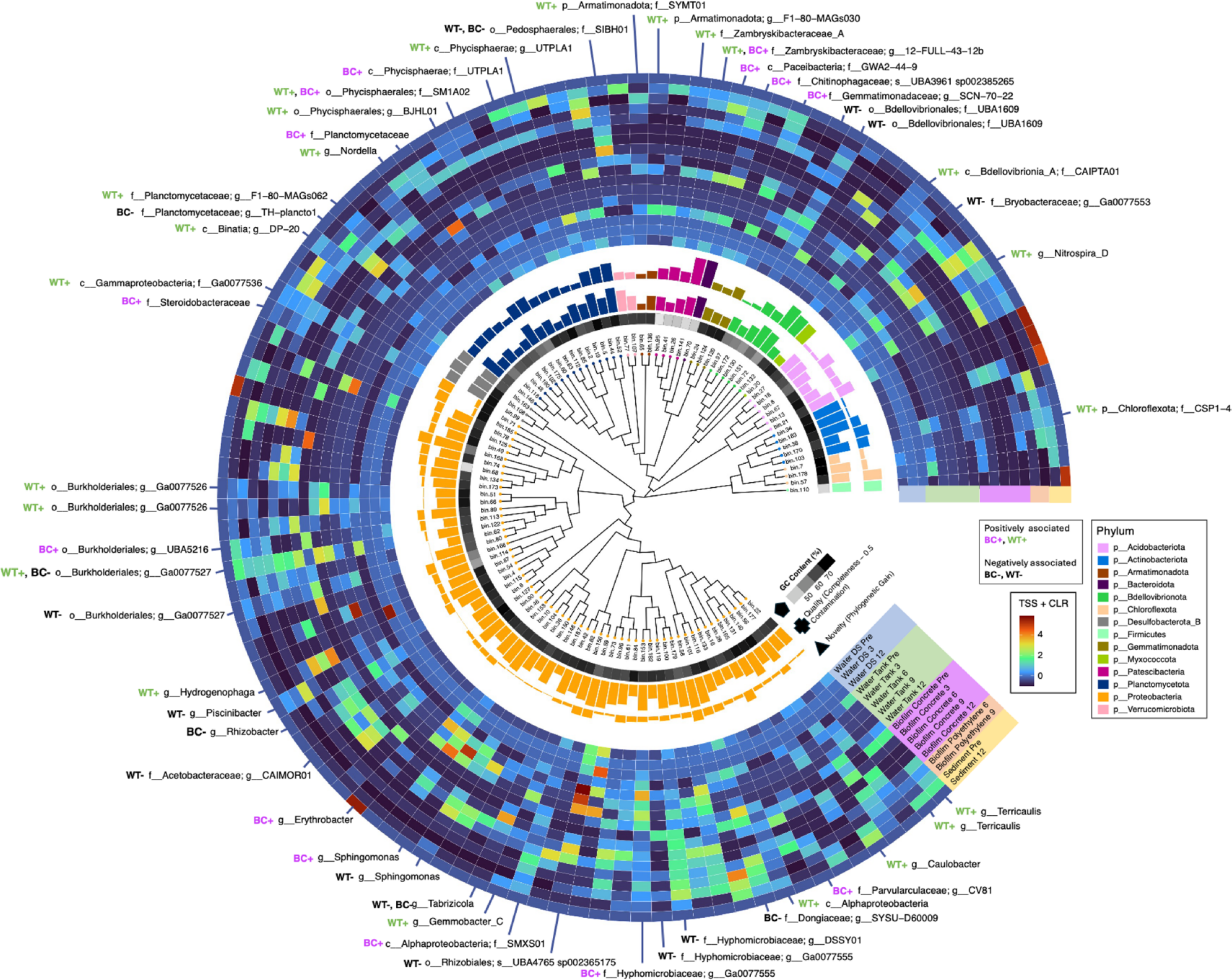
**(A)** Proportion of core OTUs belonging to different phyla level based on SILVA SSU Ref NR database release v.138 taxonomy. We have incorporated a *Time-Specific Occupancy Model* (multiple replicates for each temporal point, namely, *Pre*, *3 months*, *6 months*, and *12 months*) with details given in Supplementary Figure S2. The taxonomic coverage at different occupancies is shown in Supplementary Figures S3-S7. (**B**) The count of the number of core OTUs detected as neutral, below (selected by dispersal limitation), and above (selected by host), and classified at Phylum level, respectively. The legends are as follows: DS: Water Distribution System; T: Water Tank; C: Biofilm Concrete; and P: Biofilm Polyethylene

Across all SDWDS phases, Proteobacteria (67–92%) consistently dominated the core microbiome, accompanied by Actinobacteriota and Bacteroidota (Fig. 5A). These phyla are well-documented as versatile and resilient in DWDS environments, regardless of system design or disinfection regime [16, 82, 83]. In addition, WDS included unique phyla such as Firmicutes (2%) and Deinococcota (1%), suggesting adaptation to high disinfectant exposure, shorter residence times, and greater hydraulic variability [81, 84].

In contrast, WT exhibited higher diversity sharing 15 phyla with sediments and biofilms, while harbouring unique phyla like Desulfobacterota (0.2%) and Latescibacterota (Fig.5A). The greater diversity and unique phyla observed in tank water may be indicative of less selective pressure and a more nutrient-rich or stable environment promoting microbial diversification.

Sediment and biofilm core microbiomes overlapped significantly, sharing 16 phyla, including Myxococcota, a common microorganism in natural biofilms [85], which could be a potential microbial marker for biofilm establishment and maturation in DWDS environments.

According to their adaptation to different environments, taxa was classified as “host-selected” when specific factors influenced their distribution (Fig. 5B). For example, Cyanobacteria was classified as “host-selected” in BC and Ascomycota in PB. Considering sediment samples, Bdellovibrionota, Crenarchaeota, Cyanobacteria, Myxococcota, NB1-j, and Sumerlaeota were classified as “host-selected”. In tank water, the classification of Nitrospirota as “host-selected” may reflect its known role in nitrogen cycling and/or adaptation to specific hydraulic or nutrient conditions within DWDS [64]. Interestingly, Nitrospirota was also a “host-selected” taxa on polyethylene biofilms, several studies reported its presence on microplastics and plastisphere of diverse water and wastewater environments [86–89], these host-selected microorganisms, indicate, specialized ecological roles, potentially involved in nutrient cycling, microbial predation, and community shaping [90].

### Linking environmental variables to microbial community structure

To explore the environmental and temporal factors shaping the DWDS microbiome, we applied PERMANOVA on the Bray-Curtis, weighted and unweighted UniFrac, and Hierarchical Meta-Storms distance matrices. Significant contributions to community variation were observed for several variables (Supplementary Table S2), with the strongest influences attributed to summer, water, main distribution system, polyethylene biofilm, concrete wall, and presence of microbial contamination indicators such as total coliforms and *Escherichia coli* (high R² values (*p* < 0.05)) 

During warmer months, higher temperatures in the tank (located outside the building) and the low water consumption affected the community structure, followed by water phase and the type of material. This reinforces previous DWDS studies indicating that biofilm formation and composition are influenced by material type [58, 63]. Thus, different materials in SDWDS might affect the quality and safety of tap water. Interestingly, no indicator microorganisms were detected in polyethylene biofilm during summer whereas all tank phases tested positive for total coliforms (Supplementary_Data_Table.xlsx).

To further explore environmental associations with microbial abundance, we fitted a Generalized linear latent variable model (GLLVM) across 483 genera and multiple covariates, including system phase, distribution system, material, time and season (Supplementary Figures S8–S17). The model revealed clear associations, with like *Myxococcales* associated with sediment, summer and the 12-month time point. This bacterium is predominantly found in soils but can also inhabit aquatic systems [91]. Their consistent presence in warm and stagnant environments, and biofilms, suggests they could serve as potential indicators of microbiological shifts linked to water reduced quality.

We also evaluated the influence of water quality variables (pH, free chlorine, turbidity, HPC, and temperature) by overlaying them on ordination plots using penalized splines (Supplementary Figures S21-S22). Temperature and pH were the strongest drivers of microbial community shifts across all distance metrics (Bray-Curtis, UniFrac, Meta-Storms), particularly in tank water. Summer tank water samples (12 months) formed distinct clusters characterized by high pH and temperature. In contrast, WDS samples exhibited weaker seasonal separation, although chlorine residuals contributed to differences from low-chlorine tank samples. Unweighted UniFrac and Meta-Storms highlighted phylogenetic and functional shifts linked to pH and temperature, especially separating autumn water samples from the main DWDS at lower temperatures (14 °C).

These findings show that seasonality, temperature and pH, are the primary drivers of microbial community structure in SDWDS, with chlorine acting as a secondary factor. Overall, these results indicate that seasonality-driven changes—particularly increased temperature, pH, and reduced flow—create conditions that promote microbial restructuring and convergence between water, biofilm, and sediment phases within storage tanks. From a management perspective, this highlights storage tanks as critical control points within SDWDS, where maintaining adequate disinfectant residuals, minimizing water residence time, and preventing stagnation—especially during warmer periods—may be key strategies to limit microbial regrowth and reduce potential health risks. These findings highlight the urgent need to explicitly consider storage tanks within drinking water regulatory and monitoring frameworks [4].

## Conclusions

This study presents the first long-term, phase-resolved investigation of microbial communities, including biofilms, inside a full-scale drinking water storage tank system in Latin America. By combining 16 S rRNA amplicon sequencing with whole-genome metagenomics, we provide a comprehensive overview of taxonomic and functional dynamics across water, sediment, and biofilms formed on concrete and polyethylene surfaces over one year, addressing the need to understand how microbial communities develop and interact within SDWDS storage infrastructures.

Our findings reveal that storage tanks act as microbial reservoirs, with sediment and biofilm phases exhibiting higher diversity, temporal stability, and functional specialization compared to bulk water. Importantly, despite high-quality inlet water, seasonal shifts, particularly during summer, when water consumption is reduced, strongly influence community composition, promoting convergence across phases and enhancing functional redundancy. Biofilm maturation was associated with increased stress response mechanisms and antibiotic resistance traits, highlighting tanks as microbial reservoirs that could compromise downstream safety, raising potential concerns for drinking water safety. The successful recovery of high-quality MAGs enabled the identification of novel taxa and confirmed time- and surface-specific microbial succession patterns.

Together, these findings advance our understanding of SDWDS microbiology by demonstrating that environmental conditions within storage tanks—especially temperature, pH, and water residence time—are key drivers of microbial selection and persistence. From a management perspective, this supports the need to maintain adequate disinfectant residuals, minimize stagnation, and consider material-specific biofilm dynamics when designing and operating storage systems. Moreover, these results underscore the importance of explicitly incorporating storage tanks into drinking water regulatory and monitoring frameworks, as critical control points for safeguarding water quality.

While this study provides novel insights into the microbial ecology of full-scale storage tanks, further research is needed to assess the persistence and potential health impacts of biofilm-associated AGR genes, to explore fungal communities in greater depth, and to evaluate the effectiveness of tank management strategies across diverse climatic conditions and infrastructure designs.

## Supplementary Information

Supplementary Material 1: Supplementary_Data_Table.xlsx: Metadata associated with 16 S rRNA and whole genome shotgun metagenomics samples.

Supplementary Material 2: Supplementary_Information.docx: Supplementary material associated with this study including supplementary methods, figures, and tables.

## Data Availability

The raw 16 S rRNA sequences supporting the results of this article are available in the European Nucleotide Archive (ENA) under the project accession number PRJEB92103 (with metadata of samples given in Supplementary_Data_Table.xlsx), whilst the raw whole genome shotgun metagenomics sequences are available under project accession number PRJEB92108 (with metadata of samples given in Supplementary_Data_Table.xlsx).

## Abbreviations

16S rRNA gene: 16 S ribosomal RNA gene
ARG: Antibiotic Resistance Genes
ASVs: Amplicon sequence variants
BC: Biofilm formed over concrete
BP: Biofilm formed over polyethylene
DNA: Deoxyribonucleic acid
DWDS: Drinking Water Distribution System
HPC: Heterotrophic Plate Count
MAGs: Metagenome-assembled Genomes
OTUs: Operational Taxonomic Units
PBS: Phosphate Buffer Saline
RNA: Ribonucleic acid
S: Sediment
SDWDS: Secondary Drinking Water Distribution Systems
WDS: Water from the main water distribution system
WGS: Whole Genome Sequencing
WT: Tank water
