# Supplementary material for: Biofilm and sediment phases as key components of microbial community dynamics within secondary drinking water distribution systems
Source: BMC Microbiol. 2026 May 16;26:605. doi: 10.1186/s12866-026-05149-7 (PMC13348880; doi:10.1186/s12866-026-05149-7)
Supplement: Supplementary file 2 — Supplementary Material 2: Supplementary_Information.docx: Supplementary material associated with this study including supplementary methods, figures, and tables. [file 12866_2026_5149_MOESM2_ESM.docx]

### **Supplementary Information**

### Bioinformatics

**16S rRNA amplicon sequencing**

For amplicons samples (n=57), a total of 4,182,655 reads were obtained. Abundance tables were obtained by constructing Operational Taxonomic Units (OTUs), a proxy for species level assignment using a modified workflow [1] for pre-processing the amplicons dataset as these result in reducing the substitution error rates significantly. Briefly, the reads trimming, and filtration was performed using Sickle [2] resulting in a total of 4,148,174 reads from n=57 samples. We then used BayesHammer [3] to error correct the paired-end reads. The paired-end reads were then overlapped using PandaSeq [4] resulting in 4,058,250 reads from n=57 samples.

After having obtained consensus sequences from each sample, VSEARCH pipeline was used for [5] OTU construction.

The approach is as follows: we pool the reads from different samples together and add barcodes to keep an account of the samples these reads originate from. We then dereplicate the reads and sort them by decreasing abundance and discard singletons. In the next step, the reads are clustered, followed by removing clusters that have chimeric models built from more abundant reads (–uchime_denovo option in vsearch). A few chimeras may be missed, especially if they have parents that are absent from the reads or are present with very low abundance. Therefore, in the next step, we use a reference-based chimera filtering step (–uchime_ref option in vsearch) using a gold database (https://www.mothur.org/w/images/f/f1/Silva.gold.bacteria.zip). The original barcoded reads were matched against clean OTUs with 99% similarity.

SILVA SSU Ref NR database release v.138 [6] was used to assign taxonomy and generated the rooted phylogenetic tree (using qiime phylogeny align-to-tree-mafft-fasttree) within the QIIME2 framework [7]. Furthermore, PICRUSt2 [8] was used within the QIIME2 environment to recover KEGG enzymes and MetaCyc pathway predictions. For this purpose, we used the parameters --p-hsp-method pic --p-max-nsti 2 in qiime picrust2 full-pipeline [https://github.com/gavinmdouglas/q2-picrust2]. QIIME2 was also used to generate a final BIOM file that combined abundance information with the new taxonomy and which along with the newly phylogenetic tree, and the meta data was used for the downstream statistical analysis. Note that using OTUs based approach, we produced a final n=57 x P=5,966 OTUs abundance table with the summary statistics of OTUs per sample as [1st Quartile: 43,183; Median: 49,010; Mean: 54,206; 3rd Quartile: 69,061; and Max: 84,220]. Note that, prior to using VSEARCH pipeline, we have also tried to recover Amplicon Sequencing Variants (ASVs) using DADA2 [9] algorithm, however, the mean number of ASVs per sample were 12,428 which were lower than the mean number of OTUs per sample, and therefore, we processed the OTUs data further. From PICRUSt2, we recovered: n=57 x P=10,543 KEGG Orthologs abundance table; and a n=57 x P=489 MetaCyc pathways abundance table.

**Shotgun Metagenomics**

For a set of 21 metagenomic samples, the sequencing centre provided adapter-trimmed reads. Subsequently, these reads underwent quality trimming using Sickle [2]. This involved removing reads where the average Phred quality fell below 20 and retaining paired-end reads with a post-trimming length exceeding 50bp. This gave us a total of 409,081,828 reads from all samples. We aggregated both the forward and reverse reads and conducted a collective assembly for all samples using Megahit. The assembly was performed with the parameters --k-list 27,47,67,87 --kmin-1pass -m 0.95 --min-contig-len 1000 [10]. This gave us a total of 391,236 contigs, a total of 1,453,073,008 base pairs (bp), maximum of 1,627,611 bp, average length of 3,714 bp, and an N50 score of 6,762 bp. We then used MetaWRAP pipeline [11] and binned the contigs using three different binning algorithms i.e. metabat2 (381 bins) [12], maxbin2 (323 bins) [13], and CONCOCT (324 bins) [14]. We applied CheckM on these bins [15] to assess their completion and contamination. Within MetaWRAP framework, the bins from the three binners were consolidated, retaining bins with $\geq$50% completion and $\leq$10% contamination to give a final set of 183 bins [Metagenomic Assembled Genomes (MAGs)]. We obtained a mean genome completion of 77.87% and a mean contamination of 4.020% for bins. METABOLIC pipeline [16] was used to annotate the MAGs. This recovered several feature tables using numerous databases: KEGG [17], TIGRfam [18], Pfam [19] custom hidden Markov model (HMM) databases [20], dbCAN2 [21], and MEROPS [22]. Within the METABOLIC pipeline, the taxonomy of each MAGs was also found using the GTDB-TK [23] database. To deduce the phylogeny of the MAGs, we employed GToTree [24]. The software offers various Single Copy Genes (SCGs) sets based on the resolution of domains and the taxonomic rank of interest. Specifically, we utilized a 25-gene set for Bacteria and Archaea. To identify novel MAGs, we utilized the Genome Tree Toolkit available at <https://github.com/donovan-h-parks/GenomeTreeTk>. This involved assessing the phylogenetic gain for each MAG against the rest of the tree, with higher values potentially indicating novel species. We calculated these values for each MAG in the trees recovered using both the 25-gene Bacteria and Archaea SCGs set. Finally, reads from each sample was mapped against the MAGs to generate a coverage table of mean abundances using CoverM (<https://github.com/wwood/CoverM>).

### Statistical methods

For 16S rRNA dataset, as a pre-processing step, we selected for samples with >5000 reads, and removed typical contaminants such as *Mitochondria* and *Chloroplasts*, as well as any Operational Taxonomic Units (OTUs) that were unassigned at all levels, as per recommendations given at <https://docs.qiime2.org/2022.8/tutorials/filtering/>. For shotgun metagenomics, we have used >50% complete and <10% contaminated MAGs (dropping out one mock community sample which was used as a quality control) with a final table of 20 samples with abundances of 148 MAGs. The R’s vegan package [25] was used for alpha and beta diversity analyses. For alpha diversity, *Chao1 richness* (the estimated number of species/features in an abundance table) was used all the tables: OTU and MetaCyc (recovered using PICRUSt2 software) tables for 16S rRNA dataset; and on coverage table and Modules table (recovered using the METABOLIC software).

To visualise the samples from 16S rRNA dataset, we have used Principal Coordinate Analysis (PCoA) with different distance measures. Specifically, we have used three different measures in PCoA: (i) *Bray-Curtis distance* on the OTU abundance table to visualise the compositional changes; (ii) *Unweighted UniFrac distance* estimated using R’s Phyloseq package [26] to see changes between samples in terms of phylogeny; and (iii) *Hierarchical Meta-Storms* (HMS) [27], a recently proposed functional beta diversity distance which takes the observed KEGG Orthologs (KOs) recovered using PICRUSt2 software. In HMS, the functional beta diversity is calculated in a hierarchical fashion (with knowledge of which KEGG pathways these KOs coded from the KEGG BRITE Database). Additionally, Vegan package was also used to perform PERMANOVA analyses (using adonis2() function from R’s vegan package) to see if the microbial or functional community structures can be explained by different sources of variability. The R^2^ values from PERMANOVA explaining percentage variability between different groups are then provided under the PCoA figures.

To select the environmental parameters most strongly associated with the variance of the observed communities, we have also applied redundancy analysis (RDA) on different beta diversity distances using Vegan’s capscale() and ordistep() functions in the following set of commands: cap.env = capscale( abund_table.dist~., meta_table); mod0.env = capscale( abund_table.dist~1, meta_table); step.env=ordistep(mod0.env, scope=formula(cap.env), direction=”both”, Pin=0.1, perm.max=9999, R2scope=TRUE). step.env$anova with p<0.05 was then able to identify the subset of parameter which were later used in and additional PERMANOVA analysis. This approach has previously been used in [28]. For key parameters, we then fitted smooth surfaces of the covariates on ordination plot (PCoA in this case) using penalised splines through the function ordisurf() in R’s Vegan package. The method uses generalised additive model by regressing the covariates as C ~ S (Dim1, Dim2), where Dim1 and Dim2 are the ordination scores extracted from PCoA and S() is a spline function. We have only shown those covariates where the model fits i.e., p < 0.05. The results are shown in the supplementary material.

For 16S rRNA dataset, to identify core microbiome of each phase of the system (Water DS, Water Tank, Sediment, Biofilm Cement, and Biofilm Polyethylene), we have used the approach discussed in [29]. The approach i first ranks the OTUs using two metrics: time-specific occupancy (whether samples are grouped by different treatment groups, namely, *Pre,* *3 months*, *6 months*, *9 months*, and *12 months*); and replicate consistency (whether the OTUs are consistent across replicates in each temporal group). After ranking the OTUs, the subset of core taxa is constructed incrementally by adding highly prevalent to lowly prevalent OTUs, and then quantifying the contribution of the core subsets to beta diversity using the Bray-Curtis distance in the equation, $C=1-\frac{BC_{core}}{BC_{all}}$. The original authors have specified a threshold at which the core subset construction stops, i.e., where the addition of an OTU does not cause more than 2% increase in the explanatory value by Bray-Curtis distance. Independently, a neutral model [30] is fitted to the “S” shaped abundance-occupancy distributions inform the OTUs that are likely selected by the environment. For the core OTUs, this then divides them in three sets: those that fall outside the 95% confidence interval of the fitted model, and are inferred to be deterministically assembled, rather than neutrally selected (set 1), with those that are **above the model selected by the host environment** (represented by red colour; set 2), and those points below the model **are dispersal limited** (represented by blue colour; set 3). The taxonomy tree of the core microbiome across different time points (*Pre,* *3 months*, *6 months*, *9 months*, and *12 months*) were drawn using the R’s metacoder package [31].

To find a minimal set of features (16S rRNA and shotgun metagenomics tables) that have changed with respect to continuous predictors considered in this study, we have used CODA-LASSO regression (coda_glmnet() function) employing R’s coda4microbiome package [32]. The fitted regression is of the form $y_{i}=\beta_{0}+\beta_{1}\log\left( x_{1i} \right)+\ldots+\beta_{j}\log\left( x_{ji} \right)+\epsilon_{i}$ (for $i$-th sample and $j$-th feature, with $x_{ji}$ being the abundance of feature), and where the outcome $y_{i}$ is a continuous outcome variable. The model uses two constraints: a) all $\beta$-coefficients sum up to zero, that gives two disjoint sets of features, those that are positively associated, and those that are negatively associated with the continuous outcome; and b) the optimization function incorporates a LASSO shrinkage that forces some of the beta coefficients to go to zero, particularly those that do not have a relationship, thus enabling variable selection.

For 16S rRNA dataset, due to the availability of comprehensive meta data, to find the relationship between individual microbes and sources of variability, we have used Generalised Linear Latent Variable Model (GLLVM) [33]{Citation}. GLVMM extends the basic generalized linear model that regresses the mean abundances $\mu_{ij}$ (for $i$-th sample and $j$-th microbe) of individual microbes against the covariates $x_{i}$ by incorporating latent variables $u_{i}$ as $g\left( \mu_{ij} \right)=\eta_{ij}=\alpha_{i}+\beta_{0j}+\boldsymbol{x}_{i}^{T}\boldsymbol{\beta}_{j}+\boldsymbol{u}_{i}^{T}\boldsymbol{\theta}_{j}$, where $\boldsymbol{\beta}_{j}$ are the microbe specific coefficients associated with individual covariate (a 95% confidence interval of these whether positive or negative, and not crossing 0 boundary gives directionality with the interpretation that an increase or decrease in that particular covariate causes an increase or decrease in the abundance of the microbe), and $\boldsymbol{\theta}_{j}$ are the corresponding coefficients associated with latent variable. $\beta_{0j}$ are microbe-specific intercepts, whilst $\alpha_{i}$ are optional sample effects which can either be chosen as fixed effects or random effects. We have mainly used the binary variables in the model such as: *Water* (whether the sample is coming from the tank or the distribution system); *Biofilm* (whether the sample is coming from the tank wall, the cement coupon or the polyethylene coupon), *Sediment* (the sample consist of loose deposits from the bottom pf the tank formed over a year of the tank use), *Main Distribution* (the sample is water from the main distribution system before it enters to the tank), *Tank* (any sample taken from the tank as water, biofilm or sediment), *Concrete Wall* (cement biofilm samples taken from the wall of the tank), *Concrete Coupon* (biofilm samples coming from the concrete coupons), *Polyethylene* (biofilm samples coming from the polyethylene coupons), *Pre* (water, biofilm or sediment samples taken after a year of the tank use, immediately before cleaning for the start of the longitudinal experiment), *Three Months* (every water sample collected from the tank or the main distribution system after three months from the start of the experiment, and every biofilm sample formed over a three-month period on either cement or polyethylene coupons), *Six Months* (every water sample collected from the tank or the main distribution system after six months from the start of the experiment, and every biofilm sample formed over a six-month period on either cement or polyethylene coupons), *Nine Months* (every water sample collected from the tank or the main distribution system after nine months from the start of the experiment, and every biofilm sample formed over a nine-month period on either cement or polyethylene coupons), *Twelve Months* (every water sample collected from the main distribution system, along with every water and sediment sample collected from the tank after twelve months from the start of the experiment, and every biofilm sample formed over a twelve-month period on either cement, polyethylene coupons, or the tank wall), *Summer* (water, biofilm or sediment samples from the tank or water samples from the distribution system taken on summer season), *Autumn* (water, biofilm or sediment samples from the tank or water samples from the distribution system taken on autumn season), *Spring* (water, biofilm or sediment samples from the tank or water samples from the distribution system taken on spring season), *Winter* (water, biofilm or sediment samples from the tank or water samples from the distribution system taken on winter season), *Year 2023* (water, biofilm or sediment samples from the tank or water samples from the distribution system taken over the year 2023).

To model the distribution of individual microbes, we have used Negative Binomial distribution. Additionally, the approximation to the log-likelihood is done through Variational Approximation (VA) with final sets of parameters in glvmm() function being family = 'negative.binomial', method="VA", and control.start=list(n.init = 7, jitter.var = 0.1) seemed to fit well.


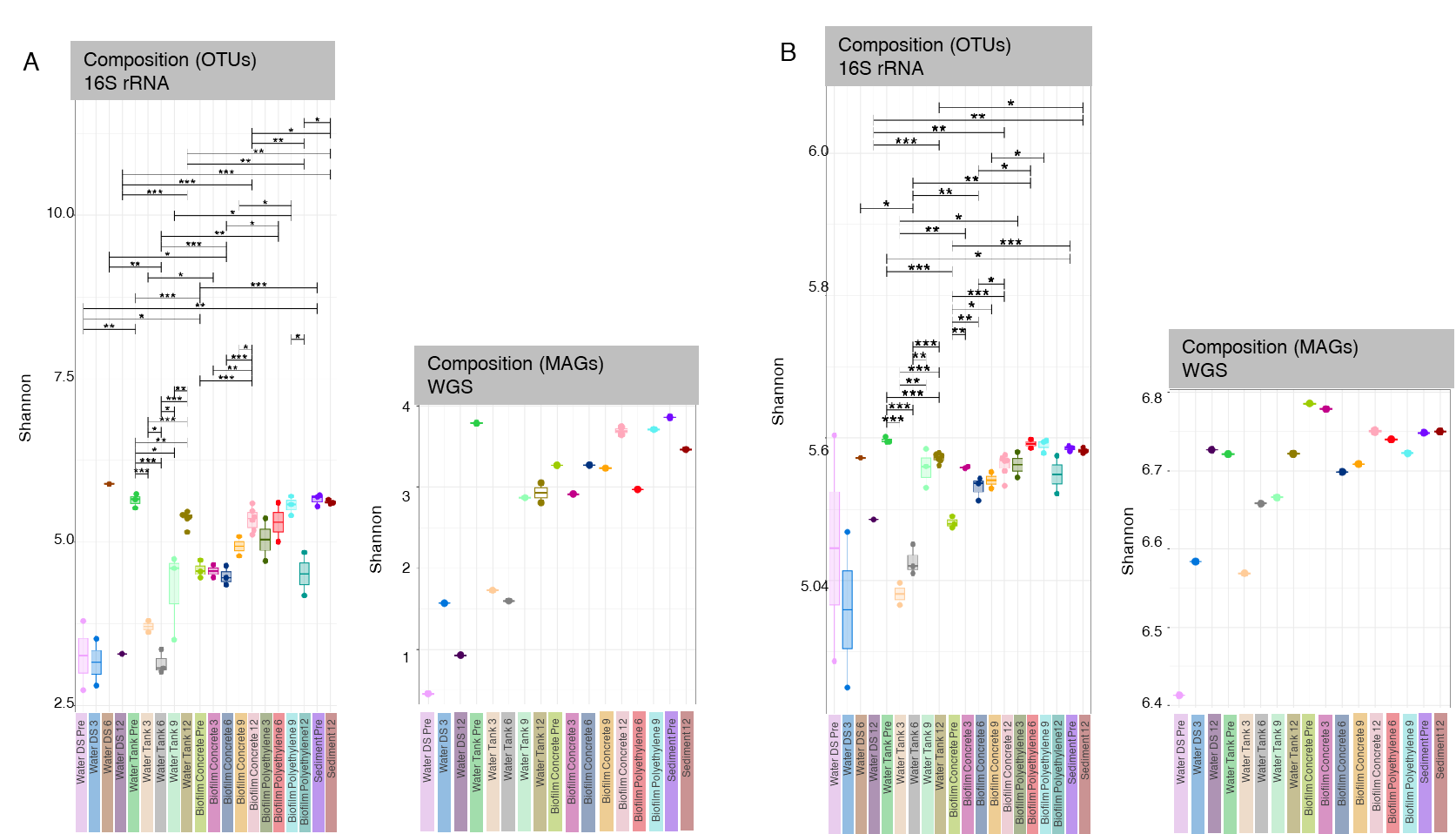


**Supplementary Figure S1**. (**A**) Shannon entropy comparison of 16S rRNA based OTUs, and MAGs returned from WGS. (**B**) The comparison of functions (MetaCyc pathway abundances returned by PICRUSt2 software on OTUs, and KEGG Sub module abundances returned by METABOLIC software for MAGs). The lines in each panel connect categories where the values are significantly different according to ANOVA with significance values as: * p < 0.05, ** p < 0.01, or *** p < 0.001.


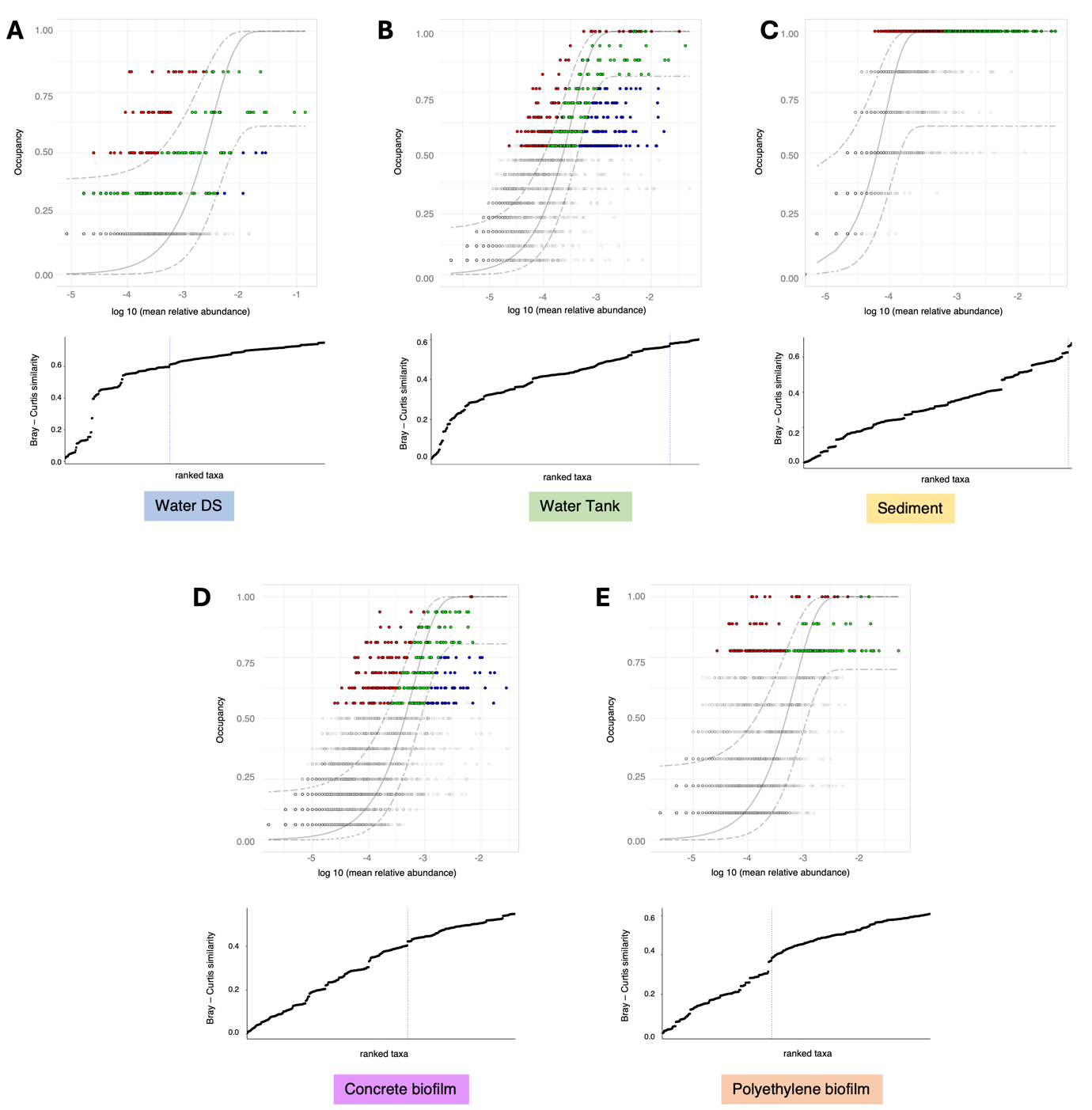


**Supplementary Figure S2**. Dynamic core microbiome inference done separately for each system using time-specific occupancy model for different systems: (A) Water Distribution System (B) Water Tank (C) Sediment (D) Concrete Biofilm and (E) Polyethylene Biofilm. The OTU rankings were obtained depending on the occupancy within these temporal groups (*Pre*, *3 months*, *6 months*, and *12 months*) as well as their replicate consistency within each temporal group. Bray-Curtis similarity is calculated for the whole dataset, and then also for only the top-ranked OTUs. The contribution of the top-ranked OTUs is divided by the total Bray-Curtis similarity to calculate a percent contribution of the core OTU set to beta diversity. The next-ranked OTU is added consecutively to find the point in the ranking at which adding one more OTUs offers diminishing returns on explanatory value for beta diversity, and is shown below the abundance-occupancy plots. Here, the vertical dotted line represents the “Last 2% decrease” criteria where OTUs are incorporated in the core subset until there is no more than 2% decrease in beta diversity. Independently, a neutral model is fitted to the dataset for each system. Combined with the core microbiome, the top plots then represent the core OTUs that are neutral, i.e., fall within the 95% interval confidence intervals shown in green, whilst non-neutral core OTUs with observed frequency above the predicted frequency from the neutral model (selected by the host) are shown in red colour, and those core OTUs with observed frequency below the predicted frequency from the neutral model (selected by dispersal limitation) are shown in green colour. All other OTUs (non-core) are greyed out.

**Supplementary Figure S3**. Taxonomic coverage tree of the Water DS samples showing **(A)** collated abundances across all occupancies (*Pre*, *3 months*, *6 months*, and *12 months*), and separately for each occupancy: **(B)** Pre, **(C)** 3 months, **(D)** 6 months, and **(E)** 12 months. The key on the right side of the trees can be interpreted as follows: the width of the bar represents the number of unique taxa and is the size of the nodes (shown on the left side of the key), whilst the colour represents the count of these taxa (shown on the right side of the key).

**Supplementary Figure S4**. Taxonomic coverage tree of the Water Tank samples showing **(A)** collated abundances across all occupancies (*Pre*, *3 months*, *6 months*, *9 months* and *12 months*), and separately for each occupancy: **(B)** Pre, **(C)** 3 months, **(D)** 6 months, **(E)** 9 months, and **(F)** 12 months. The key on the right side of the trees can be interpreted as follows: the width of the bar represents the number of unique taxa and is the size of the nodes (shown on the left side of the key), whilst the colour represents the count of these taxa (shown on the right side of the key).

**Supplementary Figure S5**. Taxonomic coverage tree of the Biofilm Concrete samples showing **(A)** collated abundances across all occupancies (*Pre*, *3 months*, *6 months*, *9 months* and *12 months*), and separately for each occupancy: **(B)** Pre, **(C)** 3 months, **(D)** 6 months, **(E)** 9 months, and **(F)** 12 months. The key on the right side of the trees can be interpreted as follows: the width of the bar represents the number of unique taxa and is the size of the nodes (shown on the left side of the key), whilst the colour represents the count of these taxa (shown on the right side of the key).

**Supplementary Figure S6**. Taxonomic coverage tree of the Biofilm Polyethylene samples showing **(A)** collated abundances across all occupancies (*3 months*, *6 months*, *9 months* and *12 months*), and separately for each occupancy: **(B)** 3 months, **(C)** 6 months, **(D)** 9 months, and **(E)** 12 months. The key on the right side of the trees can be interpreted as follows: the width of the bar represents the number of unique taxa and is the size of the nodes (shown on the left side of the key), whilst the colour represents the count of these taxa (shown on the right side of the key).

**Supplementary Figure S7**. Taxonomic coverage tree of the Sediment samples showing **(A)** collated abundances across all occupancies (*Pre*, and *12 months*), and separately for each occupancy: **(B)** Pre, and **(C)** 12 months. The key on the right side of the trees can be interpreted as follows: the width of the bar represents the number of unique taxa and is the size of the nodes (shown on the left side of the key), whilst the colour represents the count of these taxa (shown on the right side of the key).

**Supplementary Figure S8**. β-coefficients returned for individual genera (OTUs collated at genus level based on SILVA SSU Ref NR database release v.138 taxonomy) from the GLLVM procedure against the sources of variation considered in this study. Those coefficients which are positively associated with the microbial abundance of a particular species are represented in red colour whilst those that are negatively associated are represented with blue colour, respectively. Where the coefficients are non-significant, i.e., the 95% confidence interval crosses the 0 boundary, they are greyed out. All the variables are categorical variables with Yes/No [REF] as factors. REF refers to the reference against which the comparison is done, e.g., β_Autumn:Yes_ is negative (blue) for g_Actinomyces and is interpreted as “Actinomyces genus decreases in abundance for samples that are not taken in Autumn”. Β_Biofilm:Yes_ is positive (red) for g_Actinomyces and is interpreted as ““Actinomyces genus increases in abundance for samples that come from Biofilm as compared to those that do not”. The results continue on to Supplementary Figures S9-S17.

**Supplementary Figure S9**. Continuation of results from Supplementary Figure S8 for the β-coefficients returned from the GLLVM procedure.

**Supplementary Figure S10**. Continuation of results from Supplementary Figure S8 for the β-coefficients returned from the GLLVM procedure.

**Supplementary Figure S11**. Continuation of results from Supplementary Figure S8 for the β-coefficients returned from the GLLVM procedure.

**Supplementary Figure S12**. Continuation of results from Supplementary Figure S8 for the β-coefficients returned from the GLLVM procedure.

**Supplementary Figure S13**. Continuation of results from Supplementary Figure S8 for the β-coefficients returned from the GLLVM procedure.

**Supplementary Figure S14**. Continuation of results from Supplementary Figure S8 for the β-coefficients returned from the GLLVM procedure.

**Supplementary Figure S15**. Continuation of results from Supplementary Figure S8 for the β-coefficients returned from the GLLVM procedure.

**Supplementary Figure S16**. Continuation of results from Supplementary Figure S8 for the β-coefficients returned from the GLLVM procedure.

**Supplementary Figure S17**. Continuation of results from Supplementary Figure S8 for the β-coefficients returned from the GLLVM procedure.

**Supplementary Figure S18**. CODA-LASSO regression of months regressed against functional abundances for Water Tank samples using (**A**) MetaCyc pathways recovered from PICRUSt2 software on 16S rRNA based OTUs and (**B**) KEGG modules recovered form METABOLIC software for MAGs using the WGS approach. Non-zero $\beta-$coefficients returned from CODA-LASSO procedure are shown as two disjoint sets (those that are increasing with months (positive; green) and those that are decreasing with months (negative; red). The insets show prediction quality of fitting with the predictions from CODA-LASSO procedure shown on the x-axis and the actual values shown on the y-axis.

**Supplementary Figure S19**. CODA-LASSO regression of months regressed against functional abundances for Biofilm Concrete samples using (**A**) MetaCyc pathways recovered from PICRUSt2 software on 16S rRNA based OTUs and (**B**) KEGG modules recovered form METABOLIC software for MAGs using the WGS approach. Non-zero $\beta-$coefficients returned from CODA-LASSO procedure are shown as two disjoint sets (those that are increasing with months (positive; green) and those that are decreasing with months (negative; red). The insets show prediction quality of fitting with the predictions from CODA-LASSO procedure shown on the x-axis and the actual values shown on the y-axis.


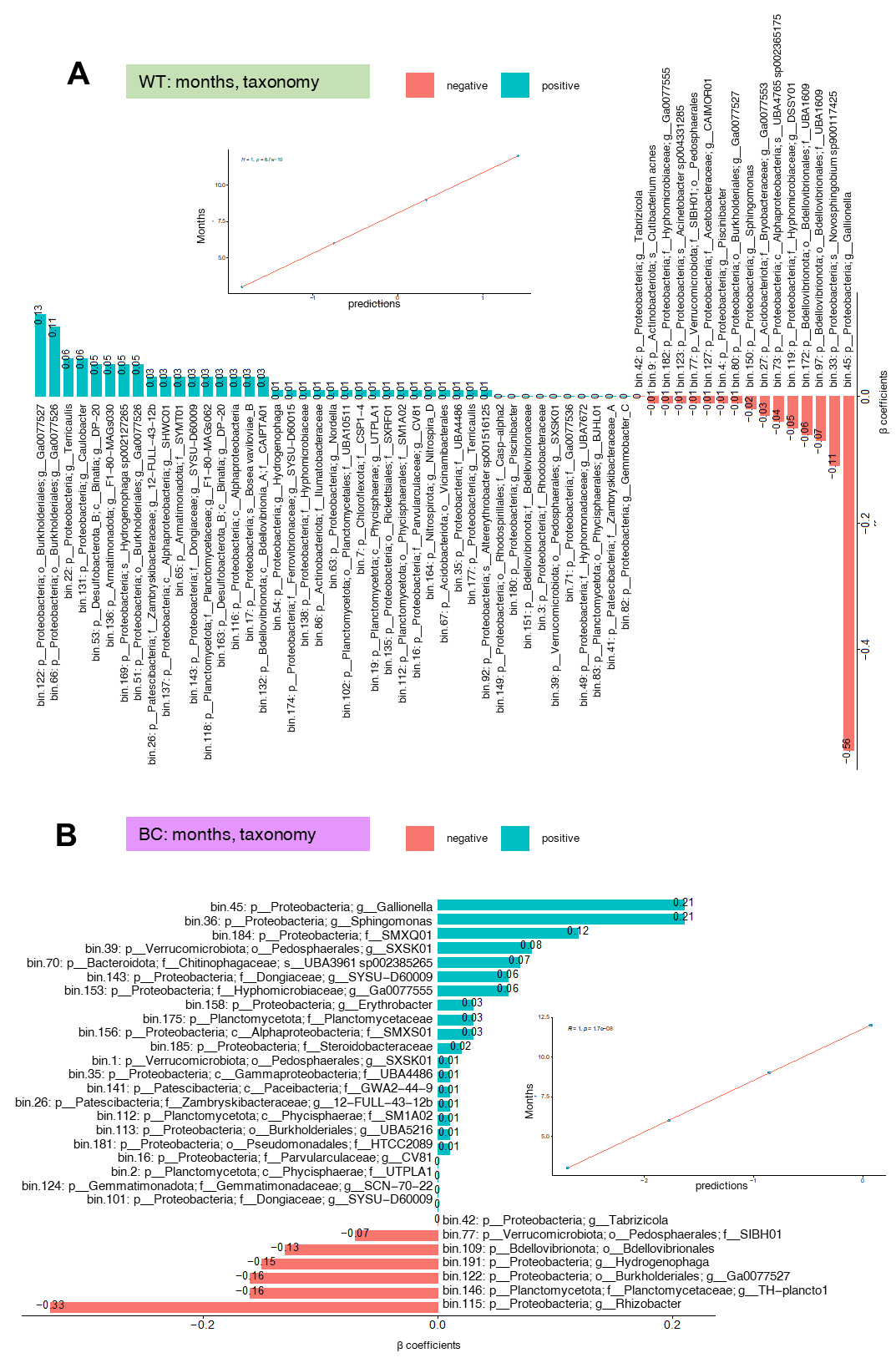


**Supplementary Figure S20**. CODA-LASSO regression of months regressed against taxonomic abundances using MAGs data with the WGS approach for (**A**) Tank Water samples and (**B**) Concrete biofilm samples. Non-zero $\beta-$coefficients returned from CODA-LASSO procedure are shown as two disjoint sets (those that are increasing with months (positive; green) and those that are decreasing with months (negative; red). The insets show prediction quality of fitting with the predictions from CODA-LASSO procedure shown on the x-axis and the actual values shown on the y-axis.

**Supplementary Figure S21**. Constrained ordination results for continuous covariates. We fitted smooth surfaces of the covariates on ordination plot (PCoA in this case) using penalised splines. The method uses generalised additive model by regressing the covariate as C ~ S(Dim1,Dim2), where Dim1 and Dim2 are the ordination scores extracted from PCoA (calculated using different beta diversity distances) and S() is a spline function. We have only shown those covariates where the model fits i.e., p < 0.05.

**Supplementary Figure S22**. Continuation of constrained ordination results for continuous covariates from Supplementary Figure S21.

**Supplementary Table S1: Summary of the 14 ARG-carrying MAGs found in concrete biofilms samples.**

| **BinID** | **GTDB-tk taxonomy** | | | | | | |
| --- | --- | --- | --- | --- | --- | --- | --- |
|  | **Domain** | **Phylum** | **Class** | **Order** | **Family** | **Genus** | **Species** |
| bin.8 | Bacteria | Acidobacteriota | Blastocatellia | RBC074 | RBC074 |  |  |
| bin.18 | Bacteria | Acidobacteriota | Acidobacteriae | Bryobacterales | Bryobacteraceae | Ga0077553 |  |
| bin.27 | Bacteria | Acidobacteriota | Acidobacteriae | Bryobacterales | Bryobacteraceae | Ga0077553 |  |
| bin.37 | Bacteria | Proteobacteria | Alphaproteobacteria | Caulobacterales | Caulobacteraceae | *Phenylobacterium* | *Phenylobacterium* sp004299445 |
| bin.46 | Bacteria | Proteobacteria | Alphaproteobacteria | Sphingomonadales | Sphingomonadaceae | *Sphingopyxis* |  |
| bin.48 | Bacteria | Planctomycetota | Planctomycetes | Planctomycetales | Planctomycetaceae | DSVQ01 |  |
| bin.58 | Bacteria | Proteobacteria | Alphaproteobacteria | Micropepsales | Micropepsaceae | SZUA-430 |  |
| bin.60 | Bacteria | Planctomycetota | Planctomycetes | Pirellulales | Pirellulaceae | UBA11363 |  |
| bin.104 | Bacteria | Proteobacteria | Alphaproteobacteria | Sphingomonadales | Sphingomonadaceae | *Sphingomonas* | *Sphingomonas* sp002403415 |
| **bin.124** | **Bacteria** | **Gemmatimonadota** | **Gemmatimonadetes** | **Gemmatimonadales** | **Gemmatimonadaceae** | **SCN-70-22** |  |
| bin.126 | Bacteria | Proteobacteria | Alphaproteobacteria | Sphingomonadales | Sphingomonadaceae | *Sphingomonas* |  |
| bin.140 | Bacteria | Proteobacteria | Alphaproteobacteria | Caulobacterales | Caulobacteraceae | *Phenylobacterium* |  |
| bin.148 | Bacteria | Proteobacteria | Alphaproteobacteria | Sphingomonadales | Sphingomonadaceae | *Sphingomonas* |  |
| bin.165 | Bacteria | Proteobacteria | Alphaproteobacteria | Caulobacterales | Caulobacteraceae | NCED01 |  |

**Supplementary Table S2**. Redundancy analysis with both selections (forward and reverse) was performed to select the most important environmental variables that explain variation in the community matrices. The initial set of variables considered are as follows (**with those selected in the final PERMANOVA models in bold case)**: system type (**main distribution**, **tank**), sample type (**water**, **biofilm**, **sediment**), months of monitoring from day 0 (**3 months**, **6 months**, 9 month, **12 months**, **Pre** (12 months of tank usage before the longitudinal experiment), tank material for biofilm formation (concrete, **polyethylene**), concrete biofilm formation (**concrete coupon**, **concrete wall**), season of sampling (**summer**, **autumn**, **winter**, **spring**), year of sampling (year 2022, **year 2023**), presence of microbial contamination indicators (**Total coliforms**, ***Escherichia coli***, ***Pseudomonas aeruginosa***).

| **Covariates** | **df** | **SS** | **R^2^** | **F** | **p** |  |
| --- | --- | --- | --- | --- | --- | --- |
| Bray-Curtis Distance | | | | | | |
| **Summer** | 1 | 23.086 | 0.11229 | 127.572 | 0.001 | *** |
| **Water** | 1 | 15.828 | 0.07699 | 87.464 | 0.001 | *** |
| **MainDistribution** | 1 | 13.809 | 0.06717 | 76.305 | 0.001 | *** |
| **Pre** | 1 | 11.888 | 0.05783 | 65.693 | 0.001 | *** |
| ***Escherichi coli*** | 1 | 11.058 | 0.05379 | 61.107 | 0.001 | *** |
| **Polyethylene** | 1 | 10.836 | 0.05271 | 59.877 | 0.001 | *** |
| **Concrete Coupon** | 1 | 0.8469 | 0.04119 | 46.796 | 0.001 | *** |
| **Winter** | 1 | 0.6765 | 0.03290 | 37.380 | 0.001 | *** |
| **Concrete Wall** | 1 | 0.6456 | 0.03140 | 35.673 | 0.001 | *** |
| **Total Coliforms** | 1 | 0.5764 | 0.02804 | 31.850 | 0.003 | ** |
| ***Pseudomonas aeruginosa*** | 1 | 0.4583 | 0.02229 | 25.323 | 0.001 | *** |
| **Year 2023** | 1 | 0.8355 | 0.04064 | 46.171 | 0.001 | *** |
| **Six Months** | 1 | 0.4474 | 0.02176 | 24.724 | 0.003 | ** |
| **Autumn** | 1 | 0.3639 | 0.01770 | 20.110 | 0.011 | * |
| Residual | 39 | 70.577 | 0.34330 |  |  |  |
| Total | 53 | 205.587 | 100.000 |  |  |  |
| Unweighted Uni-Frac | | | | | | |
| **Summer** | 1 | 17.951 | 0.15303 | 160.734 | 0.001 | *** |
| **Main Distribution** | 1 | 12.338 | 0.10518 | 110.477 | 0.001 | *** |
| **Water** | 1 | 11.102 | 0.09464 | 99.405 | 0.001 | *** |
| **Polyethylene** | 1 | 0.4627 | 0.03945 | 41.433 | 0.001 | *** |
| **Sediment** | 1 | 0.3958 | 0.03375 | 35.443 | 0.001 | *** |
| **Winter** | 1 | 0.3615 | 0.03082 | 32.373 | 0.003 | ** |
| **Total Coliforms** | 1 | 0.3461 | 0.02950 | 30.987 | 0.005 | ** |
| **Three Months** | 1 | 0.2856 | 0.02435 | 25.576 | 0.012 | * |
| **Concrete Wall** | 1 | 0.2791 | 0.02379 | 24.990 | 0.019 | * |
| **Twelve Months** | 1 | 0.2376 | 0.02026 | 21.277 | 0.026 | * |
| **Six Months** | 1 | 0.2511 | 0.02141 | 22.486 | 0.036 | * |
| *Pseudomonas aeruginosa* | 1 | 0.1776 | 0.01514 | 15.902 | 0.107 |  |
| Year 2023 | 1 | 0.1836 | 0.01565 | 16.440 | 0.088 | . |
| ***Escherichia coli*** | 1 | 0.2545 | 0.02170 | 22.788 | 0.024 | * |
| Residual | 39 | 43.556 | 0.37132 |  |  |  |
| Total | 53 | 117.300 | 100.000 |  |  |  |
| Weighted UniFrac | | | | | | |
| **Total Coliforms** | 1 | 0.022898 | 0.19862 | 212.214 | 0.001 | *** |
| **Polyethylene** | 1 | 0.009734 | 0.08443 | 90.213 | 0.001 | *** |
| **Concrete Wall** | 1 | 0.006350 | 0.05508 | 58.850 | 0.001 | *** |
| **Water** | 1 | 0.005181 | 0.04494 | 48.017 | 0.001 | *** |
| **Tank** | 1 | 0.004478 | 0.03884 | 41.503 | 0.003 | ** |
| **Summer** | 1 | 0.003908 | 0.03390 | 36.220 | 0.005 | ** |
| **Spring** | 1 | 0.003572 | 0.03098 | 33.103 | 0.011 | * |
| **Six Months** | 1 | 0.002535 | 0.02199 | 23.498 | 0.046 | * |
| **Biofilm** | 1 | 0.003357 | 0.02912 | 31.117 | 0.012 | * |
| ***Pseudomonas aeruginosa*** | 1 | 0.003124 | 0.02710 | 28.957 | 0.012 | * |
| **Year 2023** | 1 | 0.003922 | 0.03402 | 36.349 | 0.007 | ** |
| Autumn | 1 | 0.001986 | 0.01722 | 18.403 | 0.081 | . |
| Residual | 41 | 0.044238 | 0.38374 |  |  |  |
| Total | 53 | 0.115284 | 100.000 |  |  |  |
| Hierarchical Meta-Storms | | | | | | |
| **Total Coliforms** | 1 | 0.06024 | 0.17245 | 166.322 | 0.001 | *** |
| **Water** | 1 | 0.03816 | 0.10925 | 105.367 | 0.001 | *** |
| **Polyethylene** | 1 | 0.02026 | 0.05801 | 55.952 | 0.002 | ** |
| **Tank** | 1 | 0.01877 | 0.05375 | 51.837 | 0.003 | ** |
| **Concrete Wall** | 1 | 0.01471 | 0.04212 | 40.624 | 0.009 | ** |
| **Three Months** | 1 | 0.01044 | 0.02988 | 28.817 | 0.027 | * |
| Spring | 1 | 0.00865 | 0.02477 | 23.894 | 0.062 | . |
| Sediment | 1 | 0.00888 | 0.02541 | 24.509 | 0.052 | . |
| Winter | 1 | 0.00626 | 0.01792 | 17.286 | 0.129 |  |
| Autumn | 1 | 0.00554 | 0.01586 | 15.296 | 0.218 |  |
| Pre | 1 | 0.00528 | 0.01511 | 14.576 | 0.199 |  |
| Residual | 42 | 0.15211 | 0.43547 |  |  |  |
| Total | 53 | 0.34930 | 100.000 |  |  |  |
| Significance codes: 0 ‘***’ 0.001 ‘**’ 0.01 ‘*’ 0.05 ‘.’ 0.1 ‘ ’ 1 | | | | | | |

### References

[1] Schirmer M, Ijaz UZ, D’Amore R, Hall N, Sloan WT, Quince C, 2015. Insight into biases and sequencing errors for amplicon sequencing with the Illumina MiSeq platform. Nucleic Acids Res 43, e37–e37

[2] Joshi NA, Fass JN, 2011. Sickle: A sliding-window, adaptive, quality-based trimming tool for FastQ files (Version 1.33) [Software]

[3] Nikolenko SI, Korobeynikov AI, Alekseyev MA, 2013. BayesHammer: Bayesian clustering for error correction in single-cell sequencing. BMC Genomics 14, S7

[4] Masella AP, Bartram AK, Truszkowski JM, Brown DG, Neufeld JD, 2012. PANDAseq: paired-end assembler for illumina sequences. BMC Bioinformatics 13, 1–7

[5] Rognes T, Flouri T, Nichols B, Quince C, Mahé F, 2016. VSEARCH: a versatile open source tool for metagenomics. PeerJ 4, e2584. https://doi.org/10.7717/peerj.2584

[6] Quast C, Pruesse E, Yilmaz P, Gerken J, Schweer T, Yarza P, Peplies J, Glöckner FO, 2012. The SILVA ribosomal RNA gene database project: improved data processing and web-based tools. Nucleic Acids Res 41, D590–D596

[7] Bolyen E, Rideout JR, Dillon MR, Bokulich NA, Abnet CC, Al-Ghalith GA, Alexander H, Alm EJ, Arumugam M, Asnicar F, Bai Y, Bisanz JE, Bittinger K, Brejnrod A, Brislawn CJ, Brown CT, Callahan BJ, Caraballo-Rodríguez AM, Chase J, Cope EK, Da Silva R, Diener C, Dorrestein PC, Douglas GM, Durall DM, Duvallet C, Edwardson CF, Ernst M, Estaki M, Fouquier J, Gauglitz JM, Gibbons SM, Gibson DL, Gonzalez A, Gorlick K, Guo J, Hillmann B, Holmes S, Holste H, Huttenhower C, Huttley GA, Janssen S, Jarmusch AK, Jiang L, Kaehler BD, Kang KB, Keefe CR, Keim P, Kelley ST, Knights D, Koester I, Kosciolek T, Kreps J, Langille MGI, Lee J, Ley R, Liu Y-X, Loftfield E, Lozupone C, Maher M, Marotz C, Martin BD, McDonald D, McIver LJ, Melnik AV, Metcalf JL, Morgan SC, Morton JT, Naimey AT, Navas-Molina JA, Nothias LF, Orchanian SB, Pearson T, Peoples SL, Petras D, Preuss ML, Pruesse E, Rasmussen LB, Rivers A, Robeson MS, Rosenthal P, Segata N, Shaffer M, Shiffer A, Sinha R, Song SJ, Spear JR, Swafford AD, Thompson LR, Torres PJ, Trinh P, Tripathi A, Turnbaugh PJ, Ul-Hasan S, van der Hooft JJJ, Vargas F, Vázquez-Baeza Y, Vogtmann E, von Hippel M, Walters W, Wan Y, Wang M, Warren J, Weber KC, Williamson CHD, Willis AD, Xu ZZ, Zaneveld JR, Zhang Y, Zhu Q, Knight R, Caporaso JG, 2019. Reproducible, interactive, scalable and extensible microbiome data science using QIIME 2. Nat Biotechnol 37, 1091. https://doi.org/10.1038/s41587-019-0252-6

[8] Douglas GM, Maffei VJ, Zaneveld JR, Yurgel SN, Brown JR, Taylor CM, Huttenhower C, Langille MGI, 2020. PICRUSt2 for prediction of metagenome functions. Nat Biotechnol 38, 685–688

[9] Callahan BJ, McMurdie PJ, Holmes SP, 2017. Exact sequence variants should replace operational taxonomic units in marker-gene data analysis. ISME J 11, 2639–2643. https://doi.org/10.1038/ismej.2017.119

[10] Li D, Liu CM, Luo R, Sadakane K, Lam TW, 2015. MEGAHIT: An ultra-fast single-node solution for large and complex metagenomics assembly via succinct de Bruijn graph. Bioinformatics 31, 1674–1676. https://doi.org/10.1093/bioinformatics/btv033

[11] Uritskiy GV, DiRuggiero J, Taylor J, 2018. MetaWRAP—a flexible pipeline for genome-resolved metagenomic data analysis. Microbiome 6, 1–13

[12] Kang DD, Li F, Kirton E, Thomas A, Egan R, An H, Wang Z, 2019. MetaBAT 2: an adaptive binning algorithm for robust and efficient genome reconstruction from metagenome assemblies. PeerJ 7, e7359

[13] Wu Y-W, Simmons BA, Singer SW, 2016. MaxBin 2.0: an automated binning algorithm to recover genomes from multiple metagenomic datasets. Bioinformatics 32, 605–607

[14] Alneberg J, Bjarnason BS, De Bruijn I, Schirmer M, Quick J, Ijaz UZ, Lahti L, Loman NJ, Andersson AF, Quince C, 2014. Binning metagenomic contigs by coverage and composition. Nat Methods 11, 1144–1146

[15] Parks DH, Imelfort M, Skennerton CT, Hugenholtz P, Tyson GW, 2015. CheckM: assessing the quality of microbial genomes recovered from isolates, single cells, and metagenomes. Genome Res 25, 1043–1055

[16] Zhou Z, Tran PQ, Breister AM, Liu Y, Kieft K, Cowley ES, Karaoz U, Anantharaman K, 2022. METABOLIC: high-throughput profiling of microbial genomes for functional traits, metabolism, biogeochemistry, and community-scale functional networks. Microbiome 10, 33

[17] Kanehisa M, Goto S, 2000. KEGG: kyoto encyclopedia of genes and genomes. Nucleic Acids Res 28, 27–30

[18] Selengut JD, Haft DH, Davidsen T, Ganapathy A, Gwinn-Giglio M, Nelson WC, Richter AR, White O, 2007. TIGRFAMs and Genome Properties: tools for the assignment of molecular function and biological process in prokaryotic genomes. Nucleic Acids Res 35, D260–D264. https://doi.org/10.1093/nar/gkl1043

[19] Finn RD, Bateman A, Clements J, Coggill P, Eberhardt RY, Eddy SR, Heger A, Hetherington K, Holm L, Mistry J, Sonnhammer ELL, Tate J, Punta M, 2014. Pfam: the protein families database. Nucleic Acids Res 42, D222–D230. https://doi.org/10.1093/nar/gkt1223

[20] Anantharaman K, Brown CT, Hug LA, Sharon I, Castelle CJ, Probst AJ, Thomas BC, Singh A, Wilkins MJ, Karaoz U, Brodie EL, Williams KH, Hubbard SS, Banfield JF, 2016. Thousands of microbial genomes shed light on interconnected biogeochemical processes in an aquifer system. Nat Commun 7, 13219. https://doi.org/10.1038/ncomms13219

[21] Zhang H, Yohe T, Huang L, Entwistle S, Wu P, Yang Z, Busk PK, Xu Y, Yin Y, 2018. dbCAN2: a meta server for automated carbohydrate-active enzyme annotation. Nucleic Acids Res 46, W95–W101. https://doi.org/10.1093/nar/gky418

[22] Rawlings ND, Barrett AJ, Finn R, 2016. Twenty years of the MEROPS database of proteolytic enzymes, their substrates and inhibitors. Nucleic Acids Res 44, D343–D350. https://doi.org/10.1093/nar/gkv1118

[23] Chaumeil P-A, Mussig AJ, Hugenholtz P, Parks DH, 2020. GTDB-Tk: a toolkit to classify genomes with the Genome Taxonomy Database. Bioinformatics 36, 1925–1927. https://doi.org/10.1093/bioinformatics/btz848

[24] Lee MD, 2019. GToTree: a user-friendly workflow for phylogenomics. Bioinformatics 35, 4162–4164. https://doi.org/10.1093/bioinformatics/btz188

[25] Dixon P, 2003. VEGAN, a package of R functions for community ecology. J Veg Sci 14, 927–930

[26] McMurdie PJ, Holmes S, 2013. phyloseq: an R package for reproducible interactive analysis and graphics of microbiome census data. PloS One 8, e61217

[27] Zhang Y, Jing G, Chen Y, Li J, Su X, 2021. Hierarchical Meta-Storms enables comprehensive and rapid comparison of microbiome functional profiles on a large scale using hierarchical dissimilarity metrics and parallel computing. Bioinforma Adv 1, vbab003

[28] Vass M, Székely AJ, Lindström ES, Langenheder S, 2020. Using null models to compare bacterial and microeukaryotic metacommunity assembly under shifting environmental conditions. Sci Rep 10, 2455

[29] Shade A, Stopnisek N, 2019. Abundance-occupancy distributions to prioritize plant core microbiome membership. Curr Opin Microbiol 49, 50–58

[30] Burns AR, Stephens WZ, Stagaman K, Wong S, Rawls JF, Guillemin K, Bohannan BJM, 2016. Contribution of neutral processes to the assembly of gut microbial communities in the zebrafish over host development. ISME J 10, 655–664

[31] Foster ZSL, Sharpton TJ, Grünwald NJ, 2017. Metacoder: An R package for visualization and manipulation of community taxonomic diversity data. PLoS Comput Biol 13, e1005404

[32] Calle ML, Pujolassos M, Susin A, 2023. coda4microbiome: compositional data analysis for microbiome cross-sectional and longitudinal studies. BMC Bioinformatics 24, 82

[33] Niku J, Hui FKC, Taskinen S, Warton DI, 2019. gllvm: Fast analysis of multivariate abundance data with generalized linear latent variable models in r. Methods Ecol Evol 10, 2173–2182
